# Top-down/bottom-up consortia achieve robust γ-HCH degradation and reduced methanogenic contribution in wetlands

**DOI:** 10.1128/aem.01133-26

**Published:** 2026-08-28

**Authors:** Meng Liu, Xin Su, Xiaowei Huang, Xueling Yang, Yuxuan Chen, Jianming Xu, Yan He

**Affiliations:** 1 State Key Laboratory of Soil Pollution Control and Safety, College of Environmental and Resource Sciences, Zhejiang University 12377, Hangzhou, China; 2 Zhejiang Provincial Key Laboratory of Agricultural Resources and Environment, Institute of Soil and Water Resources and Environmental Science, Zhejiang University 12377 , Hangzhou, China; University of Nebraska-Lincoln, Lincoln, Nebraska, USA

**Keywords:** γ-hexachlorocyclohexane, bioremediation, organochlorine-degrading bacteria, horizontal gene transfer, methanogenesis

## Abstract

Generalist non-obligate organochlorine-degrading bacteria (ODB) play an important role in the bioremediation of γ-hexachlorocyclohexane (γ-HCH), yet their ecological roles and functional mechanisms remain underexplored. In this study, we used both “top-down” and “bottom-up” strategies to construct two functional consortia (M and Y13) based on non-obligate ODB. Both consortia demonstrated efficient degradation in sediment microcosms, reducing γ-HCH (C/C₀) to 63.0% (M) and 56.1% (Y13). Simultaneously, functional microbial consortia inoculation significantly suppressed the CO₂ reduction methanogenesis pathway compared with the non-inoculated group (*P* < 0.05), as evidenced by a marked decline in the CH₄/CO₂ ratio in inoculated groups. The well-known degradation gene *pceA* was enriched in the Y13 group, and the co-localization of *pceA*-encoding contigs with mobile genetic elements was identified. Notably, *Enterococcus* was found to have successfully colonized inoculated groups. It has strong environmental evolutionary adaptability and might acquire organochlorine-degrading genes through horizontal gene transfer under pollution stress. The broad applicability of non-obligate ODB (e.g., *Enterococcus*) makes them a promising candidate for future environmental remediation efforts, breeding new avenues of “One Health” win–win solutions in carbon reduction during pollution remediation for wetlands.

**IMPORTANCE:**

γ‑Hexachlorocyclohexane (γ‑HCH) is a representative organochlorine pesticide and a well‑known persistent organic pollutant that poses significant risks to ecosystems and human health. Microbial anaerobic degradation is a key process in the natural attenuation and engineered cleanup of γ‑HCH contamination. The effective application of obligate organohalide-respiring bacteria often requires precise management. In parallel, newly discovered non‑obligate organohalide-respiring bacteria capable of degrading γ‑HCH have emerged as promising alternatives, yet their performance and ecological interactions in realistic sediment systems remain poorly understood. This study examines the degradation mechanisms and microbial ecology of non-obligate organohalide-respiring bacterial functional consortia in complex media. The findings provide critical insights for developing effective bioaugmentation strategies for lindane‑contaminated coastal wetlands and may offer a useful framework for managing other recalcitrant halogenated pollutants in similar environments.

## INTRODUCTION

Chlorinated organic pollutants (COPs), a subset of persistent organic pollutants (POPs), are a global concern due to their high toxicity, environmental persistence, and potential for bioaccumulation (1). Among these, γ-hexachlorocyclohexane (γ-HCH, lindane) is a representative organochlorine pesticide and a well-known POP. A field survey of coastal wetlands in eastern China found that γ-HCH poses the most serious environmental risk among the detected contaminants in this region (2). Bioaugmentation, the introduction of exogenous degraders, has shown promise, with its success hinging on the acquisition and rational construction of functional microbial consortia (3, 4). Organochlorine-degrading bacteria (ODB) are broadly classified as obligate and non-obligate types based on their metabolic dependence on dehalogenation (5). Obligate ODB are more specialist, while non-obligate ODB are more generalist (6). Obligate ODB (e.g., *Dehalococcoides*) employ reductive dehalogenation as an obligate energy-conserving process, representing a high degree of metabolic specialization (7, 8). This specialization often requires specific symbiotic partners and stable redox conditions to thrive (8). In contrast, non-obligate ODB (e.g., *Geobacter*) display relatively metabolic flexibility, enabling survival in heterogeneous environments (6, 9, 10). The metabolic flexibility of non-obligate ODB might mediate the coupling between COP degradation and other biogeochemical cycling processes (e.g., methanogenesis) (11). Moreover, generalist non-obligate ODB often possess an open pangenome (characterized by high genomic diversity and abundant mobile elements) ODB (12–14). This architecture facilitates horizontal gene transfer (HGT), promoting the acquisition of catabolic genes for organochlorine degradation. Obligate ODB and non-obligate ODB are both important in COPs bioremediation. Especially, non-obligate ODB, with their broader ecological niche, are better suited for developing bioremediation strategies to address complex environmental restoration needs. However, the mechanisms underlying their function in complex environmental media remain unresolved, hindering consortium optimization for field applications.

Microbial consortia exhibit superior efficiency for pollutant degradation compared to single bacterial strains in complex environments, owing to functional redundancy and synergistic interactions (15). The “top-down” approach applies selective pressures (e.g., prolonged pollutant exposure) to steer native community assembly toward target functions, yielding self-optimized degradative microbiomes (16). Conversely, the “bottom-up” strategy designs synthetic consortia from isolated strains by programming microbial interactions (e.g., electron transfer or nutrient cycling), enabling precise control over community structure (17, 18). Top-down methods better emulate natural systems, offering the advantage of environmental adaptability; bottom-up approaches enable precise control over community composition, facilitating mechanistic insights. This duality informs emerging hybrid paradigms for consortium engineering, a key theme explored in this work. Additionally, microbial consortia, particularly those dominated by generalists, exhibit broad metabolic capabilities and are able to influence other biogeochemical processes in complex environments. Recent studies by Cheng et al. (19) have demonstrated that regulating and optimizing such consortia through conductive materials can achieve a win–win remediation strategy, facilitating γ-HCH degradation while inhibiting methane accumulation. This underscores the importance of adopting a holistic approach under the “One Health” paradigm, where the broader impacts of microbial interventions on biogeochemical cycles must be considered. The concept of One Health recognizes that human health is inextricably linked to the health of animals, plants, and the environment (20). Soil microbiomes are a cornerstone of this framework, acting as reservoirs of microbial diversity and mediating functions that directly or indirectly support all life. In this context, a microbial consortium that couples pollutant degradation with the suppression of methane (a potent greenhouse gas) offers a clear One Health solution: it remediates environmental contamination while mitigating a climate-active emission, thereby protecting both ecosystem and public health. However, current research often lacks this integrated focus, particularly regarding the mechanisms by which designed consortia function and exert influence in complex matrices like sediments.

This study employed both “top-down” and “bottom-up” microbial engineering strategies to construct functional consortia dominated by non-obligate ODB. Using sediment microcosm experiments, we systematically evaluated the degradation efficiency of γ-HCH and the microbial impact of these engineered consortia. Through integrating 16S rRNA amplicon sequencing and metagenomic analysis, we investigated (i) the impact of bioaugmentation on biogeochemical processes (in our case, γ-HCH degradation and its co-occurring process, methanogenesis) and the microbial community in wetland sediment; (ii) the colonization dynamics of introduced degraders; and (iii) the genetic mechanisms underlying γ-HCH degradation through functional annotation and metabolic pathway reconstruction. Our study aims to elucidate the colonization patterns and degradation potential of non-obligate ODB in heterogeneous sediment environments, with a focus on their metabolic plasticity and adaptive strategies, and develop a novel bioaugmentation framework, providing both theoretical and practical insights for bioremediation of complex environmental media.

## MATERIALS AND METHODS

### Microcosm experimental setup

This study designed two microbial communities through “top-down” and “bottom-up” strategies. The “top-down” strategy yielded microbial community Y13. In summary, microbial cells were extracted from samples of wetland sediment that had been polluted with COPs. γ-HCH was selected as a representative targeted pollutant. The yeast Casitones Fatty Acid (YCFA) medium with γ-HCH (1–10 mg L^−1^) was utilized for microbial amplification culture and selective acclimatization. The γ-HCH-degrading consortium Y13 was pre-enriched through 12 successive transfers, achieving ~60% degradation within 10 days at the 12th transfer, whereas no significant degradation was observed initially. 16S rRNA gene sequencing of the 12th transfer (four replicate cultures) revealed a community dominated by Firmicutes (~80%) and Actinobacteriota (~19%), with *Enterococcus* as the most predominant genus (Fig. S1). The 13th transfer was used as the top-down inoculum in this study.

The bottom-up synthetic consortium (M) was rationally constructed following function-based SynCom (synthetic community) design strategies (17, 21). Recognizing that metabolic interactions are central to community stability and function, we assembled an interacting microbial consortium comprising obligate and non-obligate organohalide-degrading bacteria, corrinoid-producing strains, and fermentative partners. The consortium comprised the obligate organohalide-respiring bacterium *Dehalococcoides mccartyi*, supported by the corrinoid-provisioning strains *Geobacter lovleyi* and *Sulfurospirillum multivorans* (9, 18), the fermentative supplier *Petrimonas sulfuriphila* PET (22), and seven isolated γ-HCH-degrading strains, several of which harbor cobalamin synthesis genes (23). A detailed list of all strains, including their source, functional rationale, and pre-culture conditions, is provided in Table S1. To ensure equitable initial representation, all component strains were combined at a 1:1 volumetric ratio of their respective cell suspensions and subsequently co-revived and co-cultivated them in YCFA medium.

The function of Y13 and M microbial community was initially verified by liquid medium-based experiments. YCFA medium was used for incubation. Three treatments were set up: (i) non-inoculated control (L-CK); (ii) inoculated with M microbial community (L-M); and (iii) inoculated with Y13 microbial community (L-Y13). Each treatment was set up with three independent biological replicates (*n* = 3). All treatments were incubated at 25°C for 10 days. γ-HCH (added as a DMSO stock solution) was supplied at a final concentration of 10 mg L⁻¹, corresponding to a DMSO concentration of 0.04% (vol/vol). Liquid samples were collected at the beginning and the end of the incubation period for the quantification of γ-HCH.

Sediment-based microcosms were set up with coastal wetland sediment and artificial seawater under anaerobic conditions. The microcosms were prepared in 250-mL anaerobic bottles with a butyl rubber stopper. A dry weight of 15 g of sediment was added to 150 mL of artificial seawater (Table S2) (24). Four treatments were conducted: (i) sterilized sediment control (CK); (ii) non-inoculated sediment control (S); (iii) sediment inoculated with M microbial community (M); and (iv) sediment inoculated with Y13 microbial community (Y13). Each treatment included three independent biological replicate microcosms (*n* = 3). Consortia M and Y13 were cultivated in parallel for the same duration under identical anaerobic conditions and medium composition. Growth was monitored by measuring optical density at 600 nm (OD_600_). Both cultures were harvested when OD_600_ exceeded 0.8 (mean values: M, 0.9; Y13, 1.3), confirming that cells were in the mid-exponential growth phase and metabolically active. For each inoculated treatment, 50 mL of bacterial culture was centrifuged, the supernatant discarded, and the cell pellet was resuspended in artificial seawater prior to addition to the microcosm system. The CK group was sterilized by autoclaving three times at 121°C for 35 minutes, with a 24-hour interval between each cycle (25). After 15 days of pre-culture, γ-HCH (added as a DMSO stock solution) was supplied at a final concentration of 10 mg kg⁻¹ dw sediment, corresponding to a DMSO concentration of 0.004% (vol/vol). The headspace was filled with nitrogen. The sediment–water mixture samples were collected at the beginning (day 0) and the end (day 30) of the incubation period for the quantification of γ-HCH, physical and chemical properties, and microbial community analysis. At the end of the incubation period, gas samples were collected.

### Analytical methods

The concentrations of γ-HCH and chlorobenzene were analyzed by an Agilent 7000D GC-8890 MS system (Agilent, Wilmington, DE) with an electron impact ion source in multiple reaction monitoring mode, as described in reference 26. The γ-HCH and chlorobenzene in liquid samples were extracted with n-hexane and dehydrated with sodium sulfate anhydrous (5). The γ-HCH and chlorobenzene in sediment-water mixture samples were extracted by a modified QuEChERS (Quick, Easy, Cheap, Effective, Rugged, and Safe) method after freeze-drying (27). Detailed information is provided in the supplemental material.

The pH of the samples was measured using a pH meter (S975 SevenExcellence, Mettler Toledo, Switzerland). Finally, CH_4_ and CO_2_ concentrations of the headspace samples were measured using gas chromatography (GC-2010 Plus, SHIMADZU, Japan).

### 16S rRNA sequencing and raw data processing

The sediment–water mixture samples of M, S, and Y13 at day 30 were used for microbial community analysis. DNA extraction was conducted using the Fast DNA SPIN Kit for soil (QBIO gene Inc., Carlsbad, CA, USA). The primer 515F/806R was used to amplify the 16S rRNA genes of the bacterial V4 region on the Illumina Nova6000 platform (Guangdong Magigene Biotechnology Co. Ltd., Guangzhou, China). The raw sequence data were processed as previously described (28). In summary, the sequences were processed using QIIME 2 (29), denoised using DADA2 (30), and annotated with the SILVA database (31).

### Shotgun metagenomic sequencing

DNA samples were collected from the M, S, and Y13 groups at day 30 for shotgun metagenomic sequencing. Following quality control, DNA samples were processed for library preparation using the ALFA-SEQ DNA Library Prep Kit. The resulting metagenomic libraries were sequenced on the Illumina or MGI platforms (Guangdong Magigene Biotechnology Co., Ltd., Guangzhou, China). Metagenomic analysis was conducted following the established genome-resolved pipeline of Huang et al. (32). Briefly, quality-filtered reads from all replicates within each treatment group (M, S, and Y13) were pooled for treatment-specific co-assembly using MEGAHIT v1.2.9. Contigs were binned to reconstruct metagenome-assembled genomes (MAGs). Open reading frames (ORFs) were predicted using Prodigal v2.6.3 (33). MAG meeting quality thresholds (>50% completeness and <10% contamination) were taxonomically classified using GTDB-Tk (34) and functionally annotated against the Kyoto Encyclopedia of Genes and Genomes (KEGG) (35) and mobileOG (36) databases. ORFs were compared to the mobileOG database using Diamond v2.0.8 (37). The MAGs of *Enterococcus* spp. (bin161, bin381, and bin436) were screened for virulence factors using the Virulence Factor Database (VFDB; https://www.mgc.ac.cn/VFs/) (37). Antibiotic resistance genes (ARGs) were annotated in both the contigs and the *Enterococcus* spp. MAGs through the Comprehensive Antibiotic Resistance Database (CARD) (38).

### Statistical analysis

16S rRNA gene amplicon sequencing data were performed to assess overall community features. 16S rRNA sequencing data were analyzed by the microeco package (39) for data preprocessing, taxa abundance analysis, alpha diversity analysis, beta diversity analysis, and network analysis. The modified stochasticity ratio (MST), a specific form of the normalized stochasticity ratio, was employed to quantitatively assess ecological stochasticity using the NST package (40). A threshold of 0.5 distinguishes deterministic assembly (MST <0.5) from stochastic assembly (MST >0.5). The average variation degree (AVD) was calculated to evaluate the stability of microbial communities, with lower AVD values indicating higher community stability (41).

Shotgun metagenomic sequencing was conducted for taxonomic profiling at the species level, differential abundance analysis, and functional annotation. Differential analysis was conducted separately for the inoculated M and Y13 groups relative to the non-inoculated S group. MaAsLin2 (Microbiome Multivariable Associations with Linear Models) was employed to identify enriched bins and enriched functions (42, 43). Further analysis was conducted on the KEGG annotation results related to organochlorine-degrading pathways, focusing specifically on 00625 (chloroalkane and chloroalkene degradation) and 00361 (chlorocyclohexane and chlorobenzene degradation). MAGs carrying typical organochlorine-degrading genes (e.g., *pceA*) were selected to analyze the distribution of these functional genes. Additional attention was also paid to the co-localization of mobile genetic elements (MGEs) and these functional genes. Based on the KEGG annotation results, the DiTing toolkit was utilized to organize and analyze annotations related to elemental cycling processes (44). PceA amino acid sequences (126 total; 57 from this study and 69 KEGG K21647 references) were aligned with MAFFT and subjected to maximum-likelihood phylogenetic analysis using PhyML + SMS on NGPhylogeny.fr, with tree visualization in iTOL. Comparative genomic analysis was performed using MAGs annotated as *Enterococcus*, whole-genome sequences of inoculated *Enterococcus* strains, and genomic information of reference strains from the NCBI database by the IPGA platform (45).

All statistical analyses were conducted on R software or OriginPro 2021 (OriginLab Corporation, Northampton).

## RESULTS

### Degradation efficiency of the microbial communities

The two microbial communities, employing either “top-down” (Y13) or “bottom-up” (M) strategies, both demonstrated effective degradation in both liquid medium and sediment medium. The degradation performance of the L-M and L-Y13 groups in the liquid medium is shown in Fig. S2. Compared with the non-inoculated control group (L-CK), the inoculated groups L-M and L-Y13 exhibited significant degradation effects (*P* < 0.05). At the end of the incubation period, the C/C₀ of γ-HCH in the L-M group was 32.7%, with chlorobenzene production reaching 87.4 ng L⁻¹. The L-Y13 group achieved nearly complete degradation, with a final C/C₀ of γ-HCH at 0.75% and a chlorobenzene concentration of 353.9 ng L⁻¹. This indicates a significant improvement in the degradation of γ-HCH to chlorobenzene by the microbial community. The degradation performance of Y13 was superior to that of M in the liquid medium (*P* < 0.05).

**Fig 1:**
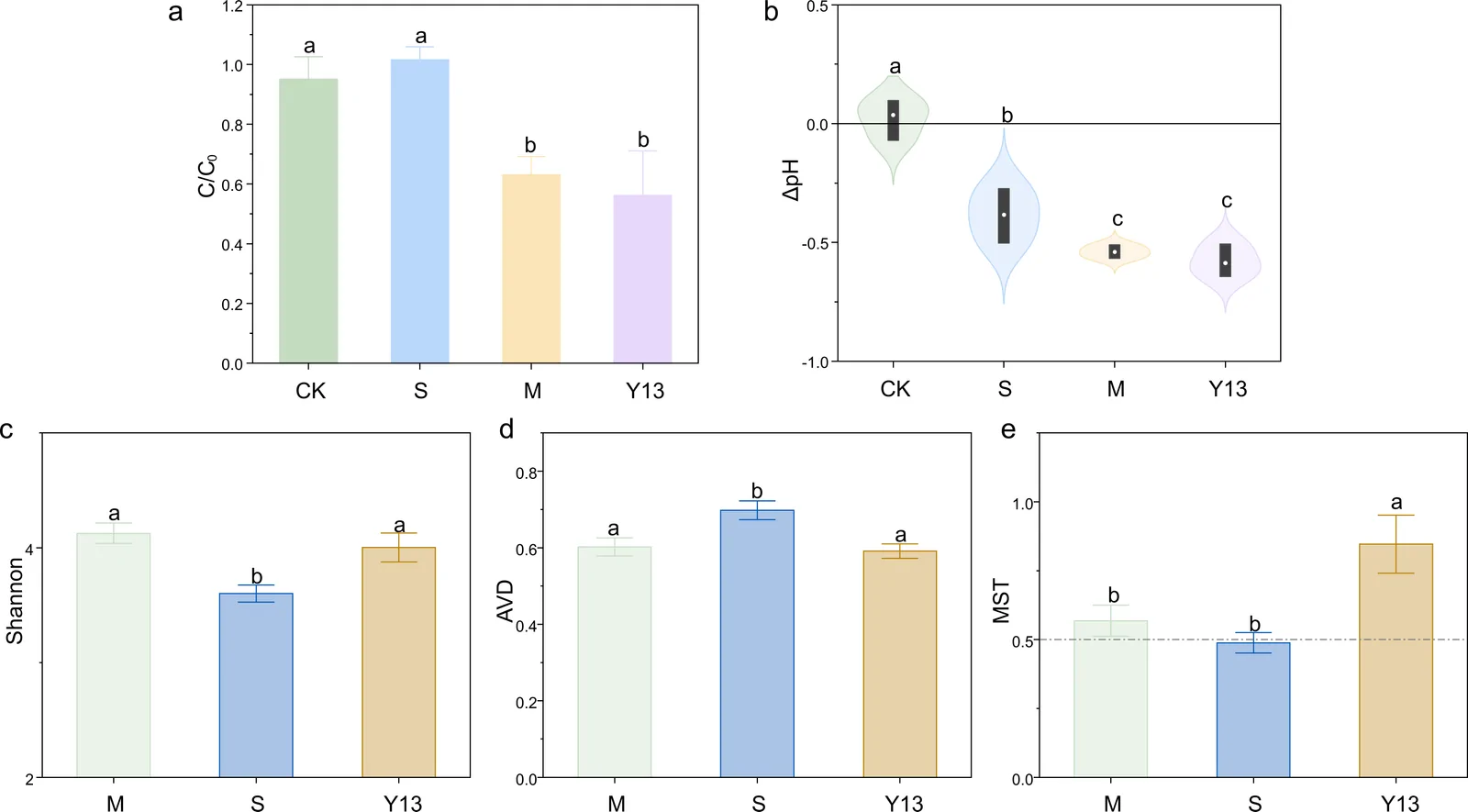
Bioaugmentation effects on γ-HCH degradation and microbial community dynamics based on 16S rRNA gene amplicon sequencing in sediment medium: (**a**) γ-HCH concentration (C/C₀), *P* = 0.01; (**b**) pH variation (ΔpH), *P* = 6.11 × 10⁻⁵; (**c**) Shannon diversity index, *P* = 0.02; (**d**) AVD, *P* = 0.03; and (**e**) MST, *P* = 0.03. *P* values were derived from one-way ANOVA comparing all groups. Different lowercase letters indicate statistically significant differences (*P* < 0.05), and groups sharing the same letter are not significantly different.

In the sediment-based system, the degradation effects of M and Y13 are presented in Fig. 1a. Compared with the S and CK groups, the inoculated M and Y13 groups exhibited significant degradation effects (*P* < 0.05). At the end of the incubation period, the C/C₀ of γ-HCH was 63.0% and 56.1% for the M and Y13 groups, respectively. However, no significant difference was observed in degradation efficiency between the M and Y13 groups.

The addition of microbial communities resulted in alterations to the physicochemical properties of the sediments. Compared to the S and CK groups, the pH reduction was significantly greater (*P* < 0.05) in the M and Y13 groups (Fig. 1b). The gas samples collected at the end of the incubation period were analyzed for CH_4_ and CO_2_ content (Fig. S4). While CH₄ concentration showed no significant difference among treatments, CO₂ concentration was significantly higher in the M and Y13 groups compared to the CK and S groups. A significant difference (*P* < 0.05) was observed in the CH_4_/CO_2_ ratio among the groups, with the M and Y13 groups exhibiting notably lower ratios compared to the CK and S groups (Fig. 6c).

### Diversity, stability, and assembly process of microbial community

Microbial inoculation significantly altered the microbial communities of the sediments. Compared to the non-inoculated sediment control (group S), the α-diversity of microbial communities was significantly increased (*P* < 0.05) in the groups inoculated with functional bacteria (groups M and Y13). Specifically, the mean Shannon index values were 3.60, 4.12, and 4.03 for groups S, M, and Y13, respectively (Fig. 1c). β-diversity analysis further revealed that the Bray–Curtis distance was significantly higher (*P* < 0.05) for group Y13 compared to groups M and S (Fig. S5). Additionally, microbial inoculation enhanced community stability, as evidenced by the lower AVD index in groups M and Y13 compared to group S (*P* < 0.05). The mean AVD index values were 0.70, 0.60, and 0.59 for groups S, M, and Y13. Overall, the addition of functional bacteria increased microbial diversity and improved community stability. Microbial inoculation influenced microbial community assembly. The results showed that group S was dominated by deterministic processes, while groups M and Y13 were dominated by stochastic processes. The mean MST values were 0.49 for group S, 0.57 for group M, and 0.85 for group Y13 (Fig. 1e). Group Y13 exhibited a significantly higher MST value (*P* < 0.05) compared to groups M and S. These findings indicate that the addition of functional microbial communities shifted the community assembly process toward stochastic dominance. Differential abundance analysis (Fig. S6) shows that *Enterococcus* is significantly enriched (*P* < 0.05) in group Y13, serving as a marker species in this group.

### Annotation of microbial species by metagenomic analysis and colonization of inoculated communities

**Fig 2:**
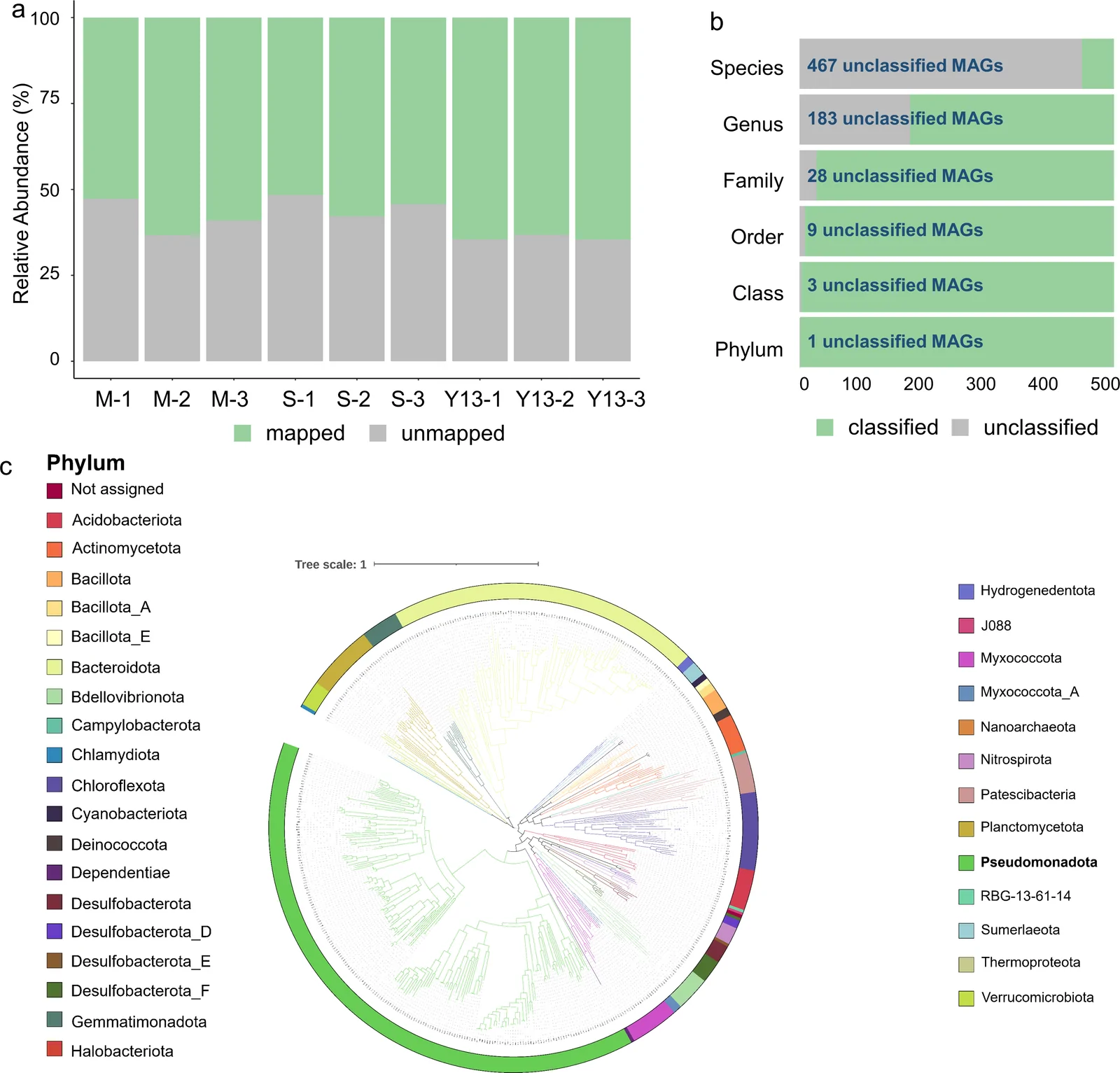
Taxonomic profiling of 520 MAGs: (**a**) sample-specific MAGs recovery rates, (**b**) novelty quantification across taxonomic ranks (unclassified = white and classified = green), and (**c**) phylum-level phylogenomic distribution.

A total of 520 high-quality MAGs were assembled through metagenomic analysis of the study. As demonstrated in Fig. 2, the proportion of reads mapping to these high-quality MAGs exceeded 50% in all samples, indicating a high level of sequence coverage and data quality. Additionally, species-level annotation of the MAGs revealed that 467 out of 520 MAGs could not be assigned to a known species, highlighting the novelty of the MAGs identified in this study and suggesting the potential existence of new species. Furthermore, a phylogenetic analysis of the 520 MAGs (Fig. 2c) revealed that the majority of these MAGs were classified into the Pseudomonadota phylum (205 MAGs), followed by Bacteroidota (109 MAGs), reflecting the dominant bacterial lineages in the studied community.

**Fig 3:**
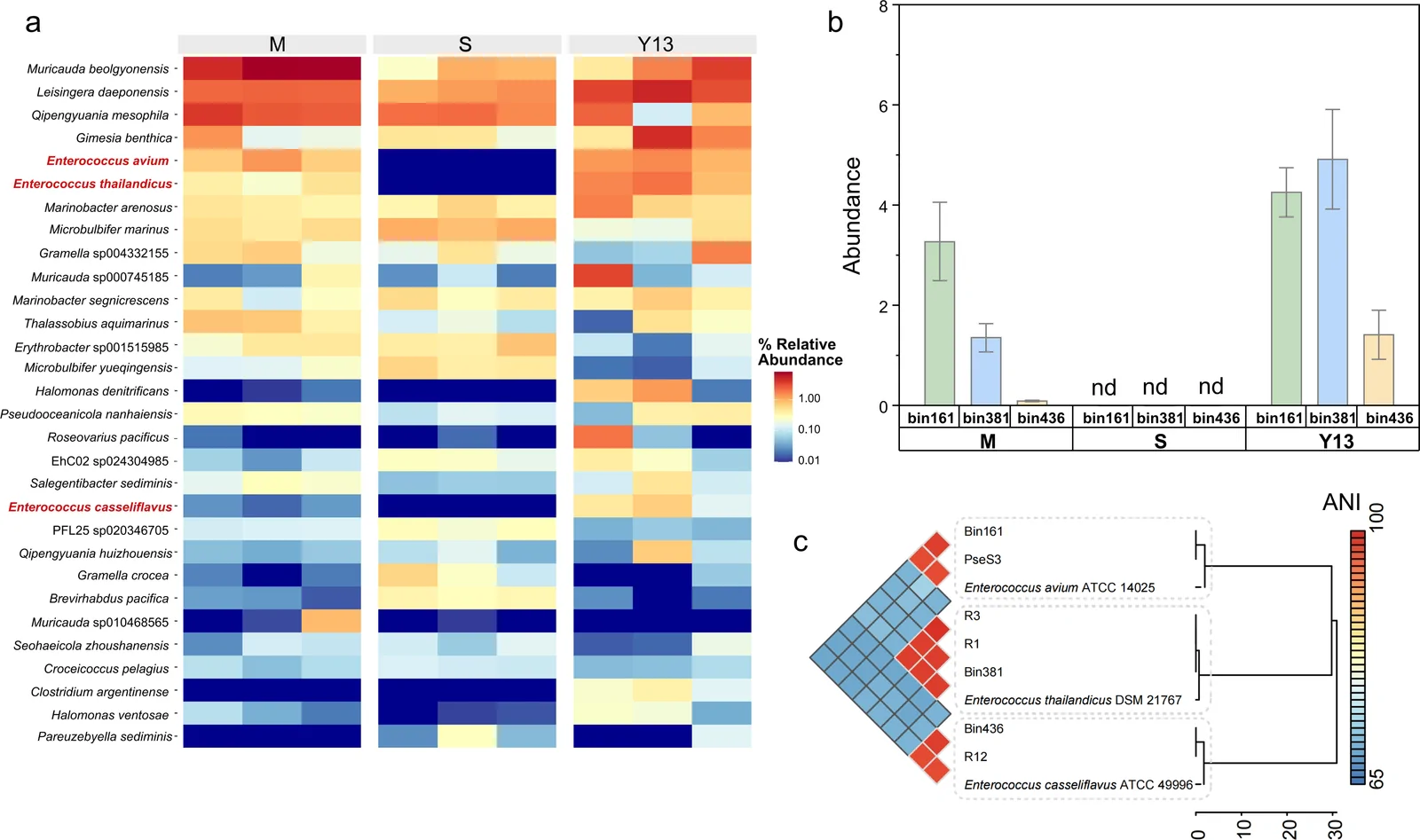
*Enterococcus* dominance and genomic features based on shotgun metagenomic data: (**a**) heatmap of top 30 species relative abundance (*Enterococcus* spp. highlighted in red), (**b**) abundance of key *Enterococcus* strains (bins 161/381/436), and (**c**) average nucleotide identity (ANI) analysis of *Enterococcus* genomes.

Tracking the inoculated strains revealed a stark difference in their fate. Except for *Enterococcus* spp., none of the other intentionally inoculated strains of community M (including *Sulfurospirillum multivorans*, *Geobacter lovleyi*, *Dehalococcoides mccartyi*, and the isolated degraders) were detected in the final sediment communities. Additionally, *Enterococcus* spp. were also identified in group Y13 sediment samples (Fig. S1), further supporting their ability to colonize and thrive. As shown in Fig. 3a, the abundance of the top 30 species at the species level revealed that *Enterococcus avium*, *Enterococcus thailandicus*, and *Enterococcus casseliflavus* exhibited relatively high abundance in both Group Y13 and Group M samples. Among the 520 MAGs, three bins (bin161, bin381, and bin436) were annotated as *Enterococcus* spp., corresponding to *Enterococcus avium*, *Enterococcus thailandicus*, and *Enterococcus casseliflavus*, respectively. The abundance of *Enterococcus* spp. in groups M, S, and Y13 is presented in Fig. 3b. Notably, bin161, bin381, and bin436 exhibited an abundance of 0 in group S, indicating the absence of these specific *Enterococcus* spp. in this group. In contrast, the mean abundance values of bin161, bin381, and bin436 were 3.27, 1.35, and 0.09, respectively, in group M, and similar values were observed in group Y13. Interestingly, bin161, bin381, and bin436 displayed higher mean abundance values (4.25, 4.91, and 1.41) in group Y13.

To further investigate the genomic characteristics of *Enterococcus* spp., the *Enterococcus* MAGs (bin161, bin381, and bin436) assembled in this study were compared with whole genomes of *Enterococcus* spp., including PseS3, R1, R3, R12, and standard strains such as *Enterococcus avium* ATCC 14025, *Enterococcus thailandicus* DSM 21767, and *Enterococcus casseliflavus* ATCC 49996, which were obtained from NCBI. Comparative genome and covariance analyses were performed, and the results are shown in Fig. S7 to S9. The covariance analysis revealed that bin436 exhibited a high degree of covariance with R12 and ATCC 49996, suggesting a close genetic relationship. The ANI values for each genome are presented in Fig. 3c. Specifically, *Enterococcus avium* ATCC 14025 shared an ANI of 98.03 with PseS3 and 98.21 with bin161. *Enterococcus thailandicus* DSM 21767 exhibited an ANI of 99.35 with R1, 99.31 with R3, and 99.35 with bin381. Similarly, *Enterococcus casseliflavus* ATCC 49996 shared an ANI of 98.24 with R12 and 98.24 with bin436. These ANI values are well above the 95% species delineation threshold, unequivocally establishing that the *Enterococcus* populations enriched in the sediment microcosms are the same species as those deliberately added to consortium M. Further IPGA comparative genome analysis of these 10 genomes revealed a total of 10,333 gene clusters, with 614 core gene clusters shared among all 10 genomes (Fig. S10 and S11). These findings provide additional evidence for the successful colonization and genomic similarity of *Enterococcus* spp. in the studied communities.

### Differential taxonomic and KEGG functional enrichment analysis

**Fig 4:**
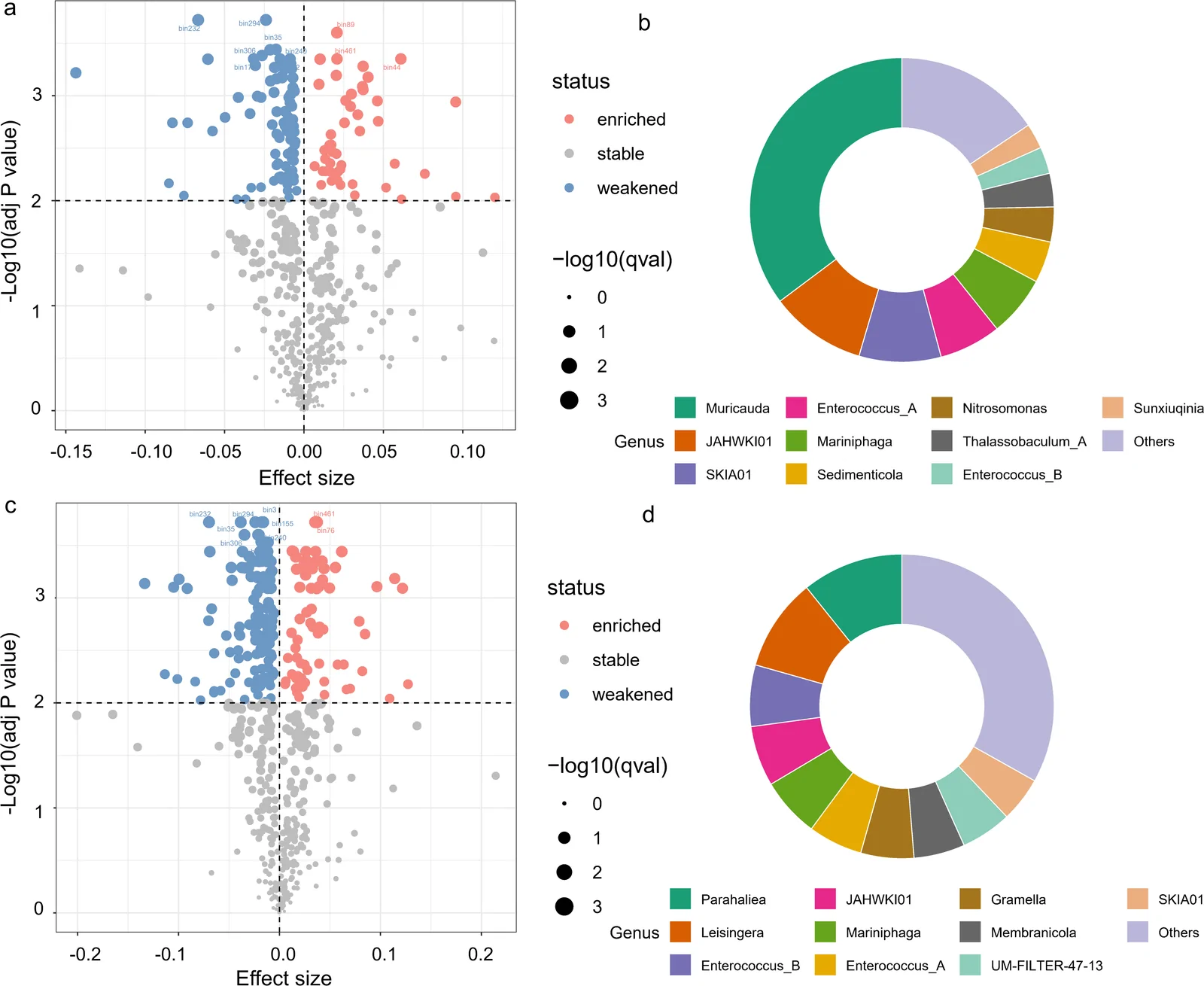
MaAsLin2-based differential abundance analysis of metagenomic species data: (**a**) M vs S (volcano plot), (**b**) M-enriched genera (effect > 0, *q* < 0.01), (**c**) Y13 vs S (volcano plot), and (**d**) Y13-enriched genera (effect > 0, *q* < 0.01).

Differential analysis of the 520 MAGs was conducted using MaAsLin2 to identify species enriched in different groups. The results are visualized in volcano plots, with Fig. 4 showing the comparison between group M and group S. A total of 47 bins were significantly enriched (effect value >0 and *q* < 0.01) in group M. Taxonomic analysis revealed that at the phylum level, Bacteroidota accounted for 46.5% of the enriched species, and Pseudomonadota represented 17.7% of the enriched species. Among the enriched species in group M, *Enterococcus avium*, *Enterococcus casseliflavus*, and *Enterococcus thailandicus* were prominent. These species belong to *Enterococcus*_A (*Enterococcus avium*) and *Enterococcus*_B (*Enterococcus thailandicus*) based on GTDB annotation, and their proportions ranked among the top 10 at the genus level. Similarly, the differential analysis between group Y13 and group S, visualized in Fig. 4, revealed 69 bins enriched in group Y13. Taxonomic composition analysis of the enriched species in group Y13 showed that Pseudomonadota was the most dominant phylum, accounting for 38.5% of the enriched species, followed by Bacteroidota (24.2%) and Bacillota (14.7%). Notably, *Enterococcus avium*, *Enterococcus casseliflavus*, and *Enterococcus thailandicus* were also enriched in group Y13, with their proportions ranking among the top 10 at the genus level. These species are classified under Bacillota, further emphasizing their ecological significance in the studied communities.

**Fig 5:**
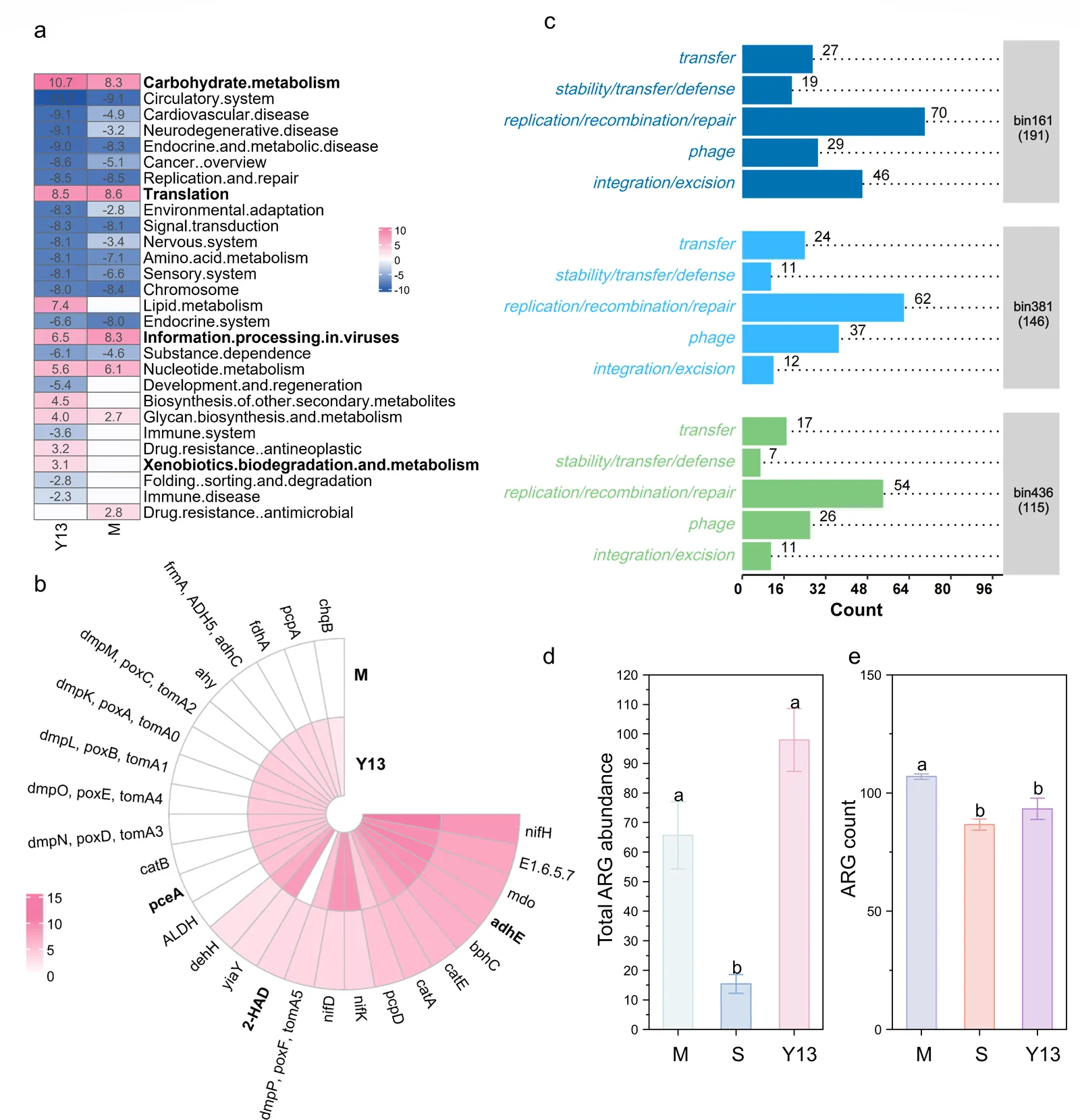
Differential functional potential: (**a**) KEGG level 2 heatmap (red/blue = enriched/depleted), (**b**) degradation pathway enrichment (ko00361 and ko00625), and (**c**) MGEs annotation of MAGs of *Enterococcus* spp. (bin161, bin381, and bin436). (**d**) Total ARG abundance (*P* = 0.002) and (**e**) ARG count (*P* = 0.008) in M, S, and Y13 groups. *P* values in panels d and e are from one-way ANOVA comparing the three groups. Different lowercase letters indicate statistically significant differences (*P* < 0.05), and groups sharing the same letter are not significantly different.

Furthermore, the results of the metagenome KEGG annotation at Level 2 were analyzed to assess functional differences between the inoculated groups (Y13 and M) and the non-inoculated group (S group). As illustrated in Fig. 5a, the functional differences were quantified using a dual-parameter system, where positive and negative values indicate enrichment (+) and weakening (−), respectively, with the magnitude reflecting the extent of change. The analysis revealed that both carbohydrate metabolism and information processing in viruses exhibited significant enrichment (*P* < 0.05) in both Y13 and M groups compared to the S group. Additionally, the Y13 group displayed notable enrichment in the degradation and metabolism of xenobiotics, biodegradation, and metabolism, suggesting enhanced detoxification capabilities in this treatment group. The KEGG-annotated orthologies (KOs) underwent differential analysis, with those related to COP degradation selected based on significant enrichment in groups Y13 and M (using group S as a reference). The enriched KOs were mapped to the KEGG pathways ko00361 and ko00625, and the results of the differential analysis are visualized in Fig. 5b as a heatmap. The findings revealed that 26 COP degradation-related genes were predominantly enriched in the Y13 group, while only 14 were enriched in the M group. Notably, the Y13 group included *pceA*, a well-known COP degradation gene, whereas the M group exhibited enrichment for *2-HAD* (EC 3.8.1.2, K01560). Phylogenetic analysis shows that the PceA-like sequences recovered from consortia M and Y13 are closely related to reference PceA sequences from known dehalogenating bacteria, with bootstrap support values exceeding 0.7 for the relevant nodes (Fig. S12).

**Fig 6:**
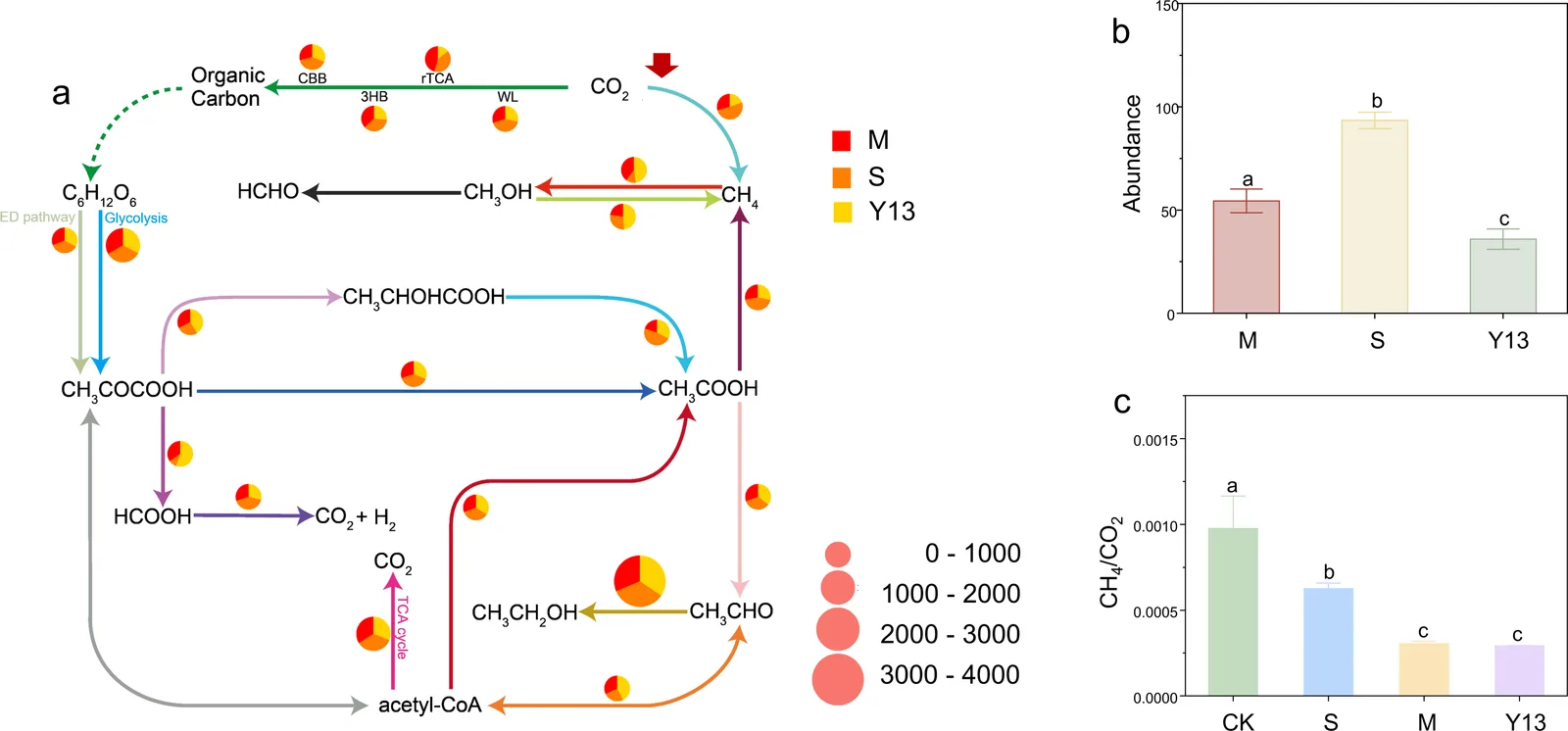
Microbial inoculation drives differential responses in carbon metabolism and methanogenesis: (**a**) functional annotation of carbon metabolic pathways (pie charts reflect relative abundance), (**b**) abundance of the CO₂-reductive methanogenesis pathway (*P* = 4.73 × 10⁻⁴, one-way ANOVA among S, M, and Y13). (**c**) CH₄/CO₂ ratio comparison (*P* = 0.003, one-way ANOVA among CK, S, M, and Y13). Different lowercase letters indicate statistically significant differences (*P* < 0.05), and groups sharing the same letter are not significantly different.

Further analysis of KEGG annotations revealed the distribution of carbon cycle pathways across the three sample groups, as illustrated in Fig. 6. This study specifically focused on the methanogenesis pathway, with emphasis on the CO_2_ reduction methanogenic pathway. The abundance of this pathway was characterized by the presence of key KEGG Orthology entries: K00200, K00201, K00202, K00203, K00205, K11260, and K00204. Statistical analysis (*P* < 0.05) showed a significant suppression of the CO_2_ reduction methanogenic pathway in the inoculated groups (M and Y13). This finding aligns with the observed trends in CH_4_/CO_2_ gas concentration ratios (Fig. 6c), indicating that bacterial addition may also have inhibited the CO_2_ reduction methanogenesis pathway.

### MGE, CARD, and VFDB annotation

The MAGs encoding the *pceA* gene were further screened and characterized, with the results presented in Fig. 5c. A total of 45 MAGs encoding the *pceA* gene were identified in this study, with species-level annotations summarized in Table S3. Among these, the highest number of MAGs (*n* = 12) belonged to the phylum Pseudomonadota. In the KEGG database, the bacteria known to encode the *pceA* gene include *Phaeobacter* sp. G2, *Sulfurospirillum multivorans*, *Sulfurospirillum halorespirans*, *Sulfurospirillum diekertiae* SL2-1, *Sulfurospirillum diekertiae* JPD-1, *Sulfurospirillum diekertiae* ACSTCE, *Desulfobaculum bizertense*, *Gottschalkiaceae bacterium* SANA, *Candidatus Contubernalis alkalaceticus*, *Dehalogenimonas etheniformans*, and Candidatus Dehalogenimonas loeffleri. However, the MAGs annotated with the *pceA* gene in this study were not represented in the above range.

Notably, a MAG assigned to *Enterococcus casseliflavus* (Bin 436) was found to encode the *pceA* gene. However, whole-genome analysis of a pure-culture isolate of *Enterococcus casseliflavus* (strain R12) did not annotate this gene, suggesting that *pceA* may have been acquired via HGT in the environmental population represented by Bin 436. To investigate the genetic context of *pceA*, the surrounding MGEs were analyzed using the mobileOG database (Table S4). Among 14 contigs from 12 MAGs where *pceA* co-localized with MGEs, the contig harboring *pceA* in *Enterococcus casseliflavus* (bin436_1) contained the highest number of associated MGEs (*n* = 11), supporting the potential role of HGT in its acquisition. The overall distribution of MGE categories across the three *Enterococcus* spp. MAGs is shown in Fig. 5c and Table S5. Bin 161 contained the highest total number of MGEs (*n* = 191), followed by Bin 381 (*n* = 146) and Bin 436 (*n* = 115). In all three MAGs, the “replication/recombination/repair” category represented the largest count of MGEs (Bin 161: 70, Bin 381: 62, and Bin 436: 57).

CARD annotation results (Fig. 5d and e) showed that the total abundance of ARGs was significantly higher in the M (65 ± 11.3) and Y13 (98 ± 10.6) groups than in the S group (15 ± 3.1) (*P* < 0.05). In contrast, the ARG count was significantly higher only in the M group (107 ± 1.2) compared to both the S (87 ± 2.4) and Y13 (93 ± 4.5) groups (*P* < 0.05). The three *Enterococcus* MAGs carried diverse but distinct sets of ARGs (Table S6). All bins shared glycopeptide resistance genes (*van*) and the efflux pump genes *efrA* and *fexA*. Notably, Bin 436 contained a relatively complete *vanC* cluster characteristic of *Enterococcus casseliflavus* and the *fosI* gene, whereas Bins 161 and 381 exhibited broader resistance to tetracyclines, aminoglycosides, and other drug classes.

The virulence factors encoded by the *Enterococcus* spp. MAGs (Bin 161, *n* = 21; Bin 381, *n* = 19; and Bin 436, *n* = 25) are listed in Tables S7 to S9. Critically, all three bins lacked complete, functional gene clusters for major toxins, such as the cytolysin (cyl) operon and gelatinase (gelE). Only the transcriptional regulator *cylR2* was identified in Bins 161 and 381, while no toxin-related genes were detected in Bin 436. All three MAGs shared virulence-associated genes related to adherence, antiphagocytosis, biofilm formation, enzymes, immune evasion, and surface protein anchoring. Distinct functional categories of each bin were as follows: Bin 161 contained genes associated with cell surface components and phagosome arrest. Bin 381 carried genes involved in iron uptake and magnesium uptake. Bin 436 featured genes related to copper uptake, invasion, nutritional factors, and secretion systems. Collectively, this virulence profile, marked by the absence of primary toxin systems but enriched with genes for adhesion, biofilm formation, and environmental stress response, strongly aligns with an environmental or commensal lifestyle rather than that of a primary pathogen. These findings suggest that the *Enterococcus* populations represented by these MAGs may have evolved to prioritize persistence and niche adaptation within complex microbial communities, such as polluted sediments, over acute host invasion.

## DISCUSSION

### Functional microbial communities efficiently degrade γ-HCH in wetland sediment

To elucidate the ecological and genomic mechanisms underlying performance disparities between the two construction strategies and to assess their respective application potential in wetland remediation, we systematically analyzed the function and composition of the consortia. In this study, two functional microbial communities constructed using the “top-down” and “bottom-up” strategies demonstrated efficient degradation capabilities in both liquid and sediment media (Fig. S1; Fig. 1a). As summarized in Table S10, the 37.0%–43.9% removal of γ-HCH in sediment media achieved within 30 days in the present study compares favorably to previously reported degradation extents under comparable conditions (e.g., 15.8% for bioaugmentation alone) (26). Moreover, this performance is also comparable to the degradation extents reported for other POPs in soil and sediment matrices, which typically range from 18.5% to 56.7% over incubation periods extending up to 120 days or 12 weeks (Table S10). Given the inherent recalcitrance of γ-HCH and the bioavailability constraints imposed by the soil/sediment matrix, the degradation observed herein represents a meaningful bioremediation outcome. Although consortium Y13 achieved a higher absolute degradation extent in sediment (43.9% vs 37.0% for M), a comparison of degradation rates normalized to liquid-phase activity revealed that consortium M retained a greater fraction of its degradative capacity upon transfer to the complex sediment matrix (18.3% vs 14.7% for Y13 Table S11), suggesting enhanced functional robustness under environmental stress.

In the pure phase system, the microbial community constructed using the “top-down” strategy exhibited significantly stronger degradation ability compared to the “bottom-up” strategy (Fig. S2). Additionally, metagenomic data from the sediment culture system indicated that the “top-down” community (Y13) showed a significant enrichment in pollutant degradation capabilities (Fig. 5a), suggesting that the “top-down” community may contain a higher proportion of functional microorganisms. However, the difference in degradation ability between the two strategies was not significant in the sediment medium (Fig. 5). This critical discrepancy suggests that in complex environments, functional output is governed not only by genetic potential but is also constrained by niche competition and metabolic burden (46, 47). In contrast, the “bottom-up” community, being simpler in composition and more controllable, retained its degradation ability even after being introduced into the sediment environment. Therefore, the “top-down” strategy plays a crucial role in isolating and obtaining functional microbial species, while the “bottom-up” strategy allows for the construction of synthetic microbial communities tailored to specific needs. In conclusion, both strategies have their own advantages and are complementary to each other. In future research, we propose integrating both strategies into a unified framework for constructing functional microbial communities, which can be used to design natural microbiomes and build synthetic microbiomes with enhanced functionality (48). Analysis of the microbial communities revealed that bioaugmentation increased diversity, enhanced stability, and shifted assembly toward stochastic dominance (Fig. 1c and d). This shift indicates that the inoculation event itself constituted a major stochastic disturbance, transiently overriding the deterministic filter imposed by the pollutant. Collectively, these changes in diversity, stability, and assembly rules demonstrate that the microbial community was jointly shaped by the stochastic inoculation of functional consortia and the deterministic selection pressure of the pollutant, a pattern consistent with established ecological theory (49).

The 30-day incubation confirmed that both consortia could initiate and sustain reductive dechlorination of γ-HCH in sediment (26), as evidenced by significant substrate removal and chlorobenzene accumulation with terminal-point evaluation (50). While this demonstrates functional proof-of-concept, it does not define the complete kinetic profile or final degradation plateau. The accumulation of chlorobenzene suggests the process may have reached a transient intermediate plateau within this timeframe. Full kinetic analysis and mass balance require future time-course studies in defined systems (51), separate from the high-chloride background of this sediment experiment. It should be noted that γ-HCH was introduced via a DMSO stock solution (final concentration 0.004% vol/vol in sediment microcosms). This concentration is 500- to 5,000-fold below reported no-effect thresholds for microbial metabolism in complex communities (Table S13) (52), and was applied identically across all groups; therefore, it is not expected to confound group-wise comparisons.

### Functional microbial communities suppress the CO₂ reduction methanogenesis pathway

Functional microbial communities suppress the CO₂ reduction methanogenesis pathway of wetland systems. In this study, CH₄ concentration showed no significant difference among treatments, while the ratio of CH₄ to CO₂ significantly declined in inoculated groups (Fig. 6c). Metagenomic functional annotation results also indicated that the CO₂ reduction to methane pathway was inhibited in groups inoculated with functional microbial communities (Fig. 6a and b). Notably, CH₄ has a global warming potential 25 times higher than CO₂ (53). From the perspective of greenhouse gas reduction, the addition of functional microbial communities contributes to suppressing CH₄ emissions, thereby mitigating the impact of global warming to some extent. It should be noted that the abiotic control (CK) was sterilized by repeated autoclaving, which may have altered sediment physicochemical properties; therefore, comparisons of absolute pH and gas values rely primarily on the non-sterilized control (group S), in which inoculation-driven differences were also significant.

This suppression may be attributed to electron competition, since the reductive dechlorination of γ-HCH and the CO₂ reduction methanogenesis pathway likely compete for common electron donors (e.g., H₂) (23). This aligns with established findings that the biodegradation of halogenated pollutants often involves complex, coupled interactions with methanogenic processes (19, 54). Furthermore, the inoculated treatments (M and Y13), which contained fermentative microorganisms and exhibited higher community diversity, experienced a more pronounced pH reduction. Given that pH is a well-established master regulator of methane-cycling microbial communities (55, 56). The observed acidification likely directly inhibited the CO₂ reduction methanogenesis pathway. This environmental shift, combined with the electron competition, effectively redirected microbial carbon flow in the system toward alternative terminal processes.

Worth mentioning, the sediments used in this study were derived from a coastal wetland, an ecosystem that is a hotspot for greenhouse gas emissions. The functional microbial communities inoculated in this study thus also achieved a dual objective of accelerating pollutant degradation while inhibiting the CO₂ reduction methanogenesis pathway, which is helpful for a win–win solution for the wetland environment, in accordance with the “One Health” goal.

### *Enterococcus* possesses traits for environmental adaptation, horizontal gene transfer, and genomic risk

Following the incorporation of the functional microbial community, *Enterococcus* spp. successfully colonized the system (Fig. 3). However, other strains of community M that were intentionally inoculated, including *Sulfurospirillum multivorans, Geobacter lovleyi, Dehalococcoides mccartyi*, and the isolated degraders, failed to colonize. It is hypothesized that the observed success is attributable to the complex sediment medium’s inherent high microbial diversity, which is postulated to reduce the likelihood of invasion by exotic species (57). *Enterococcus* is a typical generalist species, characterized by strong adaptability and the ability to colonize various environments (58, 59). Research indicates that *Enterococcus* possesses versatile metabolic capabilities, encompassing pollutant metabolism (60), carbon metabolism (61), Fe metabolism (62), etc. This study provides further evidence of the adaptability of *Enterococcus*. Among the strains selected and inoculated into sediment cultures, only *Enterococcus* spp. successfully colonized. This success is largely attributed to their metabolic flexibility, which facilitates adaptation to diverse environments.

*Enterococcus* spp. possess an open pangenome and abundant MGEs, which facilitate HGT (63, 64). The *pceA* gene (a typical functional gene for organohalide degradation) was detected in MAGs of *Enterococcus* derived from post-inoculation sediments but was absent in the complete genome sequences of the inoculated *Enterococcus* strains (PseS3, R1, R3, and R12). *PceA* was reported as involved in γ-HCH degradation (Table S12). The co-localization of *pceA* with MGEs in MAG bin436 (Fig. 5) suggests its potential acquisition via HGT. This finding is consistent with the documented dissemination of organohalide-respiring genes (e.g., *pceA*, *vcrA*) among microbial communities via HGT (12, 65). The synthetic consortium M included *Sulfurospirillum multivorans*, which could have served as a potential genetic donor for *pceA* (12). Complex, high-biomass environments like wetlands are recognized HGT hotspots (66, 67). Pollutant stress further promotes the dissemination of such adaptive genes. As facultative anaerobes, *Enterococcus* spp. are prone to HGT, especially under anaerobic conditions where gene-exchange frequencies are higher (68, 69). We acknowledge, however, that these genomic analyses address only gene presence. How pceA and related organohalide-degrading genes respond and function under contamination stress awaits further investigation using transcriptomic, proteomic, and metabolomic approaches.

ARG dissemination risk after microbial inoculation requires attention. Our results show distinct impacts: the top-down consortium (Y13) increased ARG abundance, while the bottom-up consortium (M) increased both ARG abundance and diversity (Fig. 5c and d). This difference stems from their origins. The top-down community, enriched from native sediment, mainly amplified indigenous ARGs (e.g., *Enterococcus* spp., Table S6) under pollutant stress. The bottom-up consortium introduced exogenous strains, directly adding new ARG types and increasing dissemination risk. In conditions of pollution stress, the process may also lead to the concomitant spread of antibiotic resistance genes, in addition to promoting horizontal gene transfer-mediated functional evolution (70). Thus, bioaugmentation involves a trade-off: effective degradation versus potential ARG spread. Future applications of microbial remediation technologies should strive to design consortia that not only degrade pollutants effectively but also minimize their potential to amplify and disseminate antibiotic resistance.

Considering *Enterococcus* is an opportunistic pathogen, we also assessed virulence factors in the *Enterococcus* MAGs. The analysis revealed that these MAGs lack key virulence determinants; instead, they primarily carry genes related to adhesion, biofilm formation, and environmental stress response (Tables S7 to S9). This profile strongly supports an environmental or commensal lifestyle rather than a primary pathogenic one. While most clinical enterococcal infections are attributed to *Enterococcus faecalis* or *Enterococcus faecium*, the species detected here are associated with non-clinical niches. Notably, *Enterococcus* exhibits ecological versatility: they can act as opportunistic pathogens but also display probiotic properties and are used in food fermentation, reflecting their adaptability to diverse environments (71, 72).

In conclusion, the *Enterococcus* population exemplifies the dual nature of microbial adaptations in contaminated sites: they are potential bioremediation agents carrying degradative genes (*pceA*), yet simultaneously reservoirs for mobile genetic elements and antibiotic resistance. This duality must be central to evaluating the efficacy and safety of bioaugmentation strategies in One Health frameworks.

### Non-obligate organochlorine-degrading bacteria possess broad application potential for bioremediation of complex media

In this study, a functional microbial community containing both obligate and non-obligate ODB was inoculated into sediment media. While the obligate ODB *Dehalococcoides* failed to establish, the non-obligate ODB *Enterococcus* successfully colonized and reached relatively high abundance (Fig. 3). This outcome can be attributed to the metabolic diversity and relatively open genome of non-obligate ODB. Additionally, the selected non-obligate ODB in this study were facultative anaerobes, capable of both aerobic respiration and anaerobic fermentation, enabling their survival in the presence of oxygen. In contrast, *Dehalococcoides*, a strict anaerobe, likely struggled to establish due to its inability to adapt to aerobic conditions. Furthermore, non-obligate ODB generally exhibit larger cell sizes compared to specialized degraders. For example, *Enterococcus* has a diameter of 6 μm, while *Dehalococcoides* typically ranges from 0.3 to 1 μm in diameter (73). Larger bacterial cells generally exhibit faster growth and reproduction rates (74). Additionally, it should be noted that the initial consortium was constructed by directly mixing glycerol stocks of individual strains without prior revival and verification of each strain’s viability. The OD_600_ data reported here reflect the overall growth of the enrichment culture and do not confirm that all component strains were equally revived. However, we consider that the failure of certain obligate strains to recover from glycerol stocks, together with their inability to colonize the sediment, reflects their inherently lower survival capacity. Nevertheless, the consistent HCH-degrading activity observed in the resulting consortia supports the conclusion that a functional community was established. Specialist obligate ODB (e.g., *Dehalococcoides*) often occupy narrow ecological niches, which may constrain their competitiveness and spread in complex, biodiverse environments. This specialization could also limit their engagement in HGT networks. Non-obligate degraders typically exhibit broader metabolic flexibility and ecological flexibility. This generalist lifestyle may facilitate the acquisition of catabolic genes, including those for organochlorine degradation, via HGT, thereby dynamically enhancing their adaptive potential in polluted environments (75). Consequently, in the context of successful colonization and functional persistence within heterogeneous settings like wetlands, non-obligate degraders may hold a competitive advantage. Their generalist traits and genetic plasticity likely contribute to greater versatility in complex systems. This broader applicability positions them as promising candidates for bioaugmentation strategies aimed at remediating complex environmental matrices.

### Conclusion

In our study, we designed two microbial communities through “top-down” (Y13) and “bottom-up” (M) strategies. Their degradation ability was verified by sediment-based microcosms. The C/C₀ of γ-HCH reached 63.0% and 56.1% in the M and Y13 groups, respectively. Notably, despite its lower absolute degradation, consortium M displayed higher functional robustness than Y13 when transferred from liquid to sediment. The CH_4_/CO_2_ ratio and KEGG annotation indicated a reduction of CO₂ reduction methanogenesis pathway in the inoculated groups. A total of 520 high-quality MAGs were assembled through metagenomic analysis of the study. *Enterococcus* spp. were successful to colonize and thrive. They were significantly (*P* < 0.05) enriched in M and Y13 groups. Differential KEGG functional enrichment analysis indicated that the Y13 group displayed notable enrichment in the degradation and metabolism of xenobiotics biodegradation and metabolism. *PceA* gene was also enriched in the Y13 group. The co-localization of *pceA*-encoding contigs with mobile genetic elements in 12 MAGs, including *Enterococcus casseliflavus,* potentially indicated GT-promoted dissemination. Our findings demonstrated that non-obligate ODB (e.g., *Enterococcus* in this study) possess broader application potential within complex media such as wetlands. They have generalist features. They might acquire organochlorine-degrading genes through horizontal gene transfer for better adaptation to the environment. Our results provide a novel solution for developing bioremediation strategies that couple contaminant removal with carbon reduction microbial process control under the “One Health” framework.

Genomic analysis further informs the risk–benefit profile of *Enterococcus* in bioaugmentation. Virulence factor annotation of the *Enterococcus* MAGs suggested a relatively low pathogenicity risk. However, ARG annotation highlights a potential risk of resistance dissemination following inoculation. This duality underscores that the application of *Enterococcus*-based consortia requires context-dependent risk–benefit assessment. Caution should be exercised in environmentally sensitive areas such as drinking water source zones and aquaculture regions, where ARG dissemination could pose heightened ecological and public health risks. Looking forward, we recommend that future efforts focus on strain selection or genetic engineering to decouple degradation function from resistance potential, thereby enabling safer field application.

## Data Availability

The raw sequencing reads are available under NCBI BioProject PRJNA1305168 and PRJNA1305146. The curated MAG data set (sequences, quality metrics, taxonomy, and abundance profiles) is archived in Zenodo under the following DOIs: https://doi.org/10.5281/zenodo.18217052, https://doi.org/10.5281/zenodo.19587929, and https://doi.org/10.5281/zenodo.19587929.

## SUPPLEMENTAL MATERIAL

Supplemental material aem.01133-26-s0001.docx Text S1 to S3, Fig. S1 to S13, and Tables S1 to S15.

Graphical abstract aem.01133-26-s0002.tif Visual depiction of the study highlights.

ASM does not own the copyrights to Supplemental Material that may be linked to, or accessed through, an article. The authors have granted ASM a non-exclusive, world-wide license to publish the Supplemental Material files. Please contact the corresponding author directly for reuse.
