# Supplementary material for: Top-down/bottom-up consortia achieve robust γ-HCH degradation and reduced methanogenic contribution in wetlands
Source: Appl Environ Microbiol. 2026 Aug 28;92(9):e01133-26. doi: 10.1128/aem.01133-26 (PMC13599539; doi:10.1128/aem.01133-26)
Supplement: Supplementary file 1 [file aem.01133-26-s0001.docx]

**Supplementary information (SI)**

**Top-down/bottom-up consortia achieve robust γ-HCH degradation and reduced methanogenic contribution in wetland**

Meng Liu^1^, Xin Su^1^, Xiaowei Huang^1^, Xueling Yang^1^, Yuxuan Chen^1^, Jianming Xu^1,2^, Yan He^1,2*^

*^1^ State Key Laboratory of Soil Pollution Control and Safety, College of Environmental and Resource Sciences, Zhejiang University, Hangzhou 310058, China*

*^2^ Zhejiang Provincial Key Laboratory of Agricultural Resources and Environment, Institute of Soil and Water Resources and Environmental Science, Zhejiang University, Hangzhou 31005*

**^*^Corresponding author:** Yan He; E-mail: yhe2006@zju.edu.cn; Tel.: +86-571-8898-2065; Fax: +86-571-8898-2065

# **Supplementary Texts**

Text S1 Metagenomic data processing and quality control steps

The raw sequencing reads were first subjected to quality control. Adapter sequences were removed using Cutadapt 1.17 (Martin, 2011), followed by quality control and preprocessing using fastp 0.20.0. The quality control criteria included a Phred score of at least 20 (Q20) and read lengths greater than 50 (Chen et al., 2018). The quality-approved sequences were subsequently processed for assembly, binning, and deduplication. For metagenomic assembly, MEGAHIT v1.2.9 (Li et al., 2015) was employed. Binning was performed using MetaBat2 (Kang et al., 2019), VAMB (Nissen et al., 2021), and SemiBin2 (Pan et al., 2022). The resulting metagenome-assembled genomes (MAGs) were further purified using MetaWRAP 1.3.2 (Uritskiy et al., 2018), specifically its bin refinement module. The quality of the MAGs was evaluated using CheckM2 (Chklovski et al., 2023), and MAGs meeting the criteria of completeness >50% and contamination <10% based on the Minimum Information about a Metagenome-Assembled Genome, MIMAG, standard (Bowers et al., 2017). Redundancy removal was performed using dRep v3.4.0 (Olm et al., 2017), with nucleotide similarity (ANI) >99% used as the threshold for removing redundant genomes. The relative abundance of non-redundant MAGs in each sample was calculated using CoverM 0.6.1, with parameters: "-m relative_abundance --min-read-percent-identity 0.95 --min-read-aligned-percent 0.75".

Text S2 Measurements of Iron Species and Greenhouse Gases

The concentrations of Fe^2+^ and Fe^3+^ were measured using the 1,10-phenanthroline colorimetric method by UV-Vis spectrophotometry (UV-1900, Shimadzu, Japan) at 510 nm. The changes in the ratio of Fe(III) to Fe(II) were analyzed (Fig. S2). The CK group exhibited no significant change in the Fe(III)/Fe(II) ratio before and after incubation, whereas the S, M, and Y13 groups showed significantly higher ratios (*p* < 0.05). Notably, the Y13 and M groups demonstrated greater increases in the Fe(III)/Fe(II) ratio compared to the S group.

The concentrations of CH₄ and CO₂ were measured from the headspace of the sealed microcosms. At D30, a gas-tight syringe was used to sample gas from the headspace of each serum bottle. Gas samples are transferred into sealed gas bags for analysis, and finally, gas chromatography (GC-2010 Plus SHIMADZU, Japan) is used to detect the concentrations of CO₂ and CH₄.

Text S3 Detection of γ-HCH and chlorobenzene concentration

Owing to the low aqueous solubility of γ-HCH, a stock solution was prepared in dimethyl sulfoxide (DMSO) as a carrier solvent following established procedures. Briefly, solid γ-HCH analytical standard (purity ≥ 99%) was dissolved in DMSO to yield a stock concentration of 25 g L⁻¹. For sediment microcosm experiments, a calculated volume of this DMSO-based γ-HCH stock was spiked directly into the sediment slurry under gentle mixing to achieve a nominal initial concentration of 10 mg γ-HCH per kg dry weight (dw) sediment.

For γ-HCH concentrations, the temperature program of GC-MS began at 80 ^◦^C, increased by 10 ^◦^C /min to 180 ^◦^C, held for 1 min, then increased by 5 ^◦^C /min to 200 ^◦^C, and finally increased by 20 ^◦^C /min to 280 ^◦^C. For CB concentrations, the temperature program started at 40 ^◦^C and held for 4 min, increased by 10 ^◦^C/min to 220 ^◦^C and held for 5min.

Instrument Detection Limits (IDLs) for chlorobenzene and γ-HCH were determined separately by analyzing a series of procedural blank samples. The IDL for each compound was calculated as: IDL = Mean˅blank + 3 × SD˅blank. The calculated IDLs were:1) Chlorobenzene: 0.3 ng L⁻¹; 2)γ-HCH: 0.01 ng L⁻¹. Consequently, all reported environmental concentrations in the main text have been rounded to be consistent with the precision of their respective IDL (to one decimal place for chlorobenzene and two decimal places for γ-HCH).

# **Supplementary Figures**

Fig. S1 Phylum-level composition and top 10 genera of 12^th^ transfer (Y12) consortium

Fig. S2 (a) γ-HCH concentration (C/C_0_) and (b)chlorobenzene concentrations in liquid medium-based experiment

Fig. S3. Dynamics of iron redox species in sediment systems: (a) Changes in Fe(II) (ΔFe(II)), (b) Changes in Fe(III) (ΔFe(III)), and © Fe(III)/Fe(II) ratio between control (CK, S) and inoculated groups (M, Y13). Different lowercase letters indicate statistically significant differences among treatments (*p* < 0.05, ANOVA).

Fig. S4. Headspace concentrations of CO₂ and CH₄ at the end of incubation. (a) Carbon dioxide (CO₂) concentration. (b) Methane (CH₄) concentration. Data are presented as mean ± SD (n=3). Different lowercase letters above bars indicate statistically significant differences among treatments (one-way ANOVA, p < 0.05)

Fig. S5 Difference of Bary-Curtis distance between control (S) and inoculated groups (M, Y13)

Fig. S6 LEfSe (Linear discriminant analysis Effect Size) for genus abundance of M, S and Y13 group.

Fig. S7 Synteny analysis of *Enterococcus* *avium* genomes

Fig. S8 Synteny analysis of *Enterococcus casseliflavus* genomes

Fig. S9 Synteny analysis of *Enterococcus thailandicus* genomes

Fig. S10 Curve plot of pan genome (blue) and core genome (red) cluster number of selected *Enterococcus*.

Fig. S11 The pan‐genome profile with COG annotation of selected *Enterococcus*.

Fig S12. phylogenetic tree of pceA amino acid sequences from metagenome data and KEGG dataset

Fig. S13. Proposed anaerobic degradation pathway of γ-HCH (adapted from Yuan et al., 2021) overlaid with experimental molar concentrations from this study.

# **Supplementary Tables**

Table S1. Strains used in the construction of the bottom-up synthetic consortium (M)

| **Consortium Role** | **Species/Strain Designation** | **Source / Origin** | **Key Functional Trait(s)** | **Cultivation Medium / Conditions (Pre-inoculation)** |
| --- | --- | --- | --- | --- |
| **Obligate** | *Dehalococcoides mccartyi* strain CG1 | Kindly provided by Prof. Shanquan Wang, Sun Yat-sen University. | Degradation; requires exogenous corrinoids. | Mineral salt medium, anaerobic |
| **Non-obligate** | *Geobacter lovleyi* strain LYY | Kindly provided by Prof. Shanquan Wang, Sun Yat-sen University. | Degradation; Corrinoid biosynthesis and provision to *D. mccartyi*. | Mineral salt medium, anaerobic |
| **Non-obligate** | *Sulfurospirillum multivorans* strain 12446 | Kindly provided by Prof. Yi Yang, Chinese Academy of Sciences. | Degradation; Corrinoid biosynthesis and provision to *D. mccartyi*. | Mineral salt medium, anaerobic |
| **Non-obligate** | *Petrimonas sulfuriphila* PET | Originally isolated and characterized in our previous study (Liu et al., 2025), and maintained in our laboratory culture collection. | Degradation; cobalamin synthesis gene cluster; fermentative production of electron donors. | YCFA medium, anaerobic |
| **Non-obligate** | *Robertmurraya* sp. CYTO | Originally isolated and characterized in our previous study (Liu et al., 2025), and maintained in our laboratory culture collection. | γ-HCH degradation. | YCFA medium, anaerobic |
| **Non-obligate** | *Hungatella* sp. CloS1 | Originally isolated and characterized in our previous study (Liu et al., 2025), and maintained in our laboratory culture collection. | γ-HCH degradation; putative cobalamin synthesis. | YCFA medium, anaerobic |
| **Non-obligate** | *Enterococcus avium* PseS3 | Originally isolated and characterized in our previous study (Liu et al., 2025), and maintained in our laboratory culture collection. | γ-HCH degradation. | YCFA medium, anaerobic |
| **Non-obligate** | *Enterococcus thailandicus* R1 | Newly isolated in this study as part of our ongoing research (PRJNA1308729, Liu et al., in preparation) | γ-HCH degradation. | YCFA medium, anaerobic |
| **Non-obligate** | *Enterococcus thailandicus* R3 | Newly isolated in this study as part of our ongoing research (PRJNA1308731, Liu et al., in preparation) | γ-HCH degradation. | YCFA medium, anaerobic |
| **Non-obligate** | *Enterococcus casseliflavus* R12 | Newly isolated in this study as part of our ongoing research (PRJNA1308735, Liu et al., in preparation) | γ-HCH degradation. | YCFA medium, anaerobic |

Table S2. Artificial Seawater Composition from ATCC medium: 1661 M13 Verrucomicrobium medium

| **Component** | **Concentration (g L^-1^)** | **Cl⁻ Contribution (mmol L⁻¹)** |
| --- | --- | --- |
| **NaCl** | 23.477 | 401.7 |
| **MgCl_2_** | 4.981 | 104.6 |
| **Na_2_SO_4_** | 3.917 |  |
| **CaCl_2_** | 1.102 | 19.9 |
| **KCl** | 0.664 | 8.9 |
| **NaHCO_3_** | 0.192 |  |
| **KBr** | 0.006 |  |
| **H_3_BO_3_** | 0.026 |  |
| **SrCl_2_** | 0.024 | 0.3 |
| **NaF** | 0.003 |  |
| **Total Cl^-^** |  | 535.4 |

Table S3 Annotation of species encoding MAGs of the *pce*A gene

| **ID** | **Phylum** | **Class** | **Order** | **Family** | **Genus** | **Species** |
| --- | --- | --- | --- | --- | --- | --- |
| bin155 | p__Chloroflexota | c__Anaerolineae | o__Anaerolineales | f__UBA4823 | g__PFL25 | s__ |
| bin482 | p__Bacteroidota | c__Bacteroidia | o__Bacteroidales | f__F082 | g__BM524 | s__ |
| bin12 | p__Pseudomonadota | c__Alphaproteobacteria | o__MPNO01 | f__UBA2964 | g__WHUE01 | s__ |
| bin58 | p__Bacteroidota | c__Kapabacteria | o__OLB7 | f__OLB7 | g__ | s__ |
| bin239 | p__Pseudomonadota | c__Alphaproteobacteria | o__Rhizobiales | f__Xanthobacteraceae | g__Pseudolabrys | s__ |
| bin17 | p__Chloroflexota | c__Anaerolineae | o__Aggregatilineales | f__UBA3940 | g__UBA3940 | s__ |
| **bin436** | **p__Bacillota** | **c__Bacilli** | **o__Lactobacillales** | **f__Enterococcaceae** | **g__Enterococcus_D** | **s__Enterococcus_D casseliflavus** |
| bin519 | p__Pseudomonadota | c__Alphaproteobacteria | o__Rhizobiales | f__NORP267 | g__JAGRLG01 | s__ |
| bin464 | p__Verrucomicrobiota | c__Verrucomicrobiae | o__Opitutales | f__Verruco-01 | g__Oceanipulchritudo | s__ |
| bin278 | p__Nitrospirota | c__Thermodesulfovibrionia | o__UBA6902 | f__UBA6902 | g__BMS3Abin09 | s__ |
| bin344 | p__Pseudomonadota | c__Gammaproteobacteria | o__Chromatiales | f__Sedimenticolaceae | g__Sedimenticola | s__ |
| bin220 | p__Pseudomonadota | c__Alphaproteobacteria | o__Rhodobacterales | f__Rhodobacteraceae | g__Leisingera | s__Leisingera daeponensis |
| bin75 | p__Bacillota_E | c__Symbiobacteriia | o__Symbiobacteriales | f__Symbiobacteriaceae | g__ | s__ |
| bin191 | p__Chloroflexota | c__Anaerolineae | o__Anaerolineales | f__UBA4823 | g__DSOS01 | s__ |
| bin21 | p__Bacteroidota | c__Bacteroidia | o__NORP286 | f__NORP286 | g__ | s__ |
| bin148 | p__Pseudomonadota | c__Alphaproteobacteria | o__Rhizobiales | f__Stappiaceae | g__Stappia | s__ |
| bin355 | p__Chloroflexota | c__Anaerolineae | o__Promineofilales | f__Promineofilaceae | g__JAAYYY01 | s__ |
| bin84 | p__Pseudomonadota | c__Alphaproteobacteria | o__Rhodobacterales | f__Rhodobacteraceae | g__Leisingera | s__Leisingera daeponensis |
| bin511 | p__Planctomycetota | c__Phycisphaerae | o__Sedimentisphaerales | f__Anaerohalosphaeraceae | g__VRUF01 | s__ |
| bin250 | p__Bacteroidota | c__Kapabacteria | o__OLB7 | f__OLB7 | g__ | s__ |
| bin178 | p__Nitrospirota | c__Thermodesulfovibrionia | o__Thermodesulfovibrionales | f__UBA6898 | g__G020347665 | s__ |
| bin186 | p__Acidobacteriota | c__Vicinamibacteria | o__Bin61 | f__SMYC01 | g__ | s__ |
| bin392 | p__Bacteroidota | c__Bacteroidia | o__CAILMK01 | f__JAAYUY01 | g__ | s__ |
| bin63 | p__Chloroflexota | c__Anaerolineae | o__Anaerolineales | f__UBA4823 | g__PFL25 | s__PFL25 sp020346705 |
| bin409 | p__Bacteroidota | c__Ignavibacteria | o__Ignavibacteriales | f__Melioribacteraceae | g__GCA-2746605 | s__ |
| bin244 | p__Chloroflexota | c__Anaerolineae | o__Anaerolineales | f__E44-bin32 | g__E44-bin32 | s__ |
| bin275 | p__Pseudomonadota | c__Alphaproteobacteria | o__Rhodobacterales | f__Rhodobacteraceae | g__Maritimibacter | s__Maritimibacter harenae |
| bin193 | p__Desulfobacterota | c__Desulfobulbia | o__Desulfobulbales | f__Desulfocapsaceae | g__Desulfopila | s__ |
| bin210 | p__Chloroflexota | c__Anaerolineae | o__Aggregatilineales | f__Ch27 | g__Ch27 | s__ |
| bin504 | p__Pseudomonadota | c__Alphaproteobacteria | o__Rhodobacterales | f__Rhodobacteraceae | g__EhC02 | s__EhC02 sp024304985 |
| bin240 | p__Verrucomicrobiota | c__Verrucomicrobiae | o__Verrucomicrobiales | f__DEV007 | g__JAACDM01 | s__ |
| bin430 | p__Bacillota_A | c__Clostridia | o__Tissierellales | f__MES13 | g__ | s__ |
| bin212 | p__Nitrospirota | c__Thermodesulfovibrionia | o__UBA6902 | f__UBA6902 | g__ | s__ |
| bin81 | p__Pseudomonadota | c__Gammaproteobacteria | o__Pseudomonadales | f__IMCC2047 | g__JAJFKB01 | s__ |
| bin139 | p__Acidobacteriota | c__Vicinamibacteria | o__Bin61 | f__SMYC01 | g__ | s__ |
| bin228 | p__Chloroflexota | c__Anaerolineae | o__Anaerolineales | f__UBA11579 | g__UBA11579 | s__ |
| bin241 | p__Pseudomonadota | c__Alphaproteobacteria | o__SMXQ01 | f__SMXQ01 | g__WHSO01 | s__ |
| bin272 | p__Chloroflexota | c__Anaerolineae | o__Aggregatilineales | f__UBA3940 | g__JAFGOY01 | s__ |
| bin136 | p__Chloroflexota | c__Anaerolineae | o__Anaerolineales | f__UBA4823 | g__ | s__ |
| bin479 | p__Bacillota_A | c__Clostridia | o__Clostridiales | f__Clostridiaceae | g__Clostridium_J | s__Clostridium_J argentinense |
| bin456 | p__Bacillota_E | c__Symbiobacteriia | o__Symbiobacteriales | f__Symbiobacteriaceae | g__ | s__ |
| bin90 | p__Nitrospirota | c__Thermodesulfovibrionia | o__UBA6902 | f__UBA6902 | g__BMS3Abin09 | s__ |
| bin458 | p__Nitrospirota | c__Thermodesulfovibrionia | o__UBA6902 | f__UBA6902 | g__BMS3Abin09 | s__ |
| bin420 | p__Chloroflexota | c__Anaerolineae | o__Aggregatilineales | f__UBA3940 | g__JAFGOY01 | s__ |
| bin454 | p__Pseudomonadota | c__Alphaproteobacteria | o__Sphingomonadales | f__J090 | g__J090 | s__ |

Table S4 Co-localization of the *pceA* gene with MGE

| **Bin ID** | **Contigs ID** | **MGE ID** | **MGE** |
| --- | --- | --- | --- |
| bin239 | bin239_55 | mobileOG_000025669 | phage |
| **bin436** | bin436_1 | mobileOG_000004963 | replication/recombination/repair |
| **bin436** | bin436_1 | mobileOG_000025591 | replication/recombination/repair |
| **bin436** | bin436_1 | mobileOG_000010271 | replication/recombination/repair |
| **bin436** | bin436_1 | mobileOG_000006919 | stability/transfer/defense |
| **bin436** | bin436_1 | mobileOG_000010340 | replication/recombination/repair |
| **bin436** | bin436_1 | mobileOG_000025910 | replication/recombination/repair |
| **bin436** | bin436_1 | mobileOG_000003125 | stability/transfer/defense |
| **bin436** | bin436_1 | mobileOG_000009002 | replication/recombination/repair |
| **bin436** | bin436_1 | mobileOG_000015198 | transfer |
| **bin436** | bin436_1 | mobileOG_000024559 | replication/recombination/repair |
| **bin436** | bin436_1 | mobileOG_000016652 | replication/recombination/repair |
| bin464 | bin464_30 | mobileOG_000003177 | replication/recombination/repair |
| bin239 | bin239_43 | mobileOG_000001847 | replication/recombination/repair |
| bin239 | bin239_43 | mobileOG_000001848 | replication/recombination/repair |
| bin239 | bin239_43 | mobileOG_000001843 | transfer |
| bin63 | bin63_105 | mobileOG_000026112 | replication/recombination/repair |
| bin504 | bin504_65 | mobileOG_000024870 | replication/recombination/repair |
| bin504 | bin504_65 | mobileOG_000024660 | replication/recombination/repair |
| bin430 | bin430_13 | mobileOG_000004966 | replication/recombination/repair |
| bin430 | bin430_13 | mobileOG_000010271 | replication/recombination/repair |
| bin430 | bin430_13 | mobileOG_000008090 | stability/transfer/defense |
| bin430 | bin430_13 | mobileOG_000008784 | phage |
| bin430 | bin430_13 | mobileOG_000013220 | phage |
| bin81 | bin81_7 | mobileOG_000025855 | phage |
| bin81 | bin81_7 | mobileOG_000025998 | phage |
| bin81 | bin81_7 | mobileOG_000000139 | phage |
| bin81 | bin81_7 | mobileOG_000004201 | integration/excision |
| bin228 | bin228_228 | mobileOG_000018884 | transfer |
| bin136 | bin136_79 | mobileOG_000015458 | replication/recombination/repair |
| bin479 | bin479_2 | mobileOG_000001312 | integration/excision |
| bin479 | bin479_2 | mobileOG_000014530 | replication/recombination/repair |
| bin12 | bin12_163 | mobileOG_000025515 | replication/recombination/repair |
| bin90 | bin90_58 | mobileOG_000010161 | replication/recombination/repair |
| bin479 | bin479_32 | mobileOG_000015268 | transfer |
| bin479 | bin479_32 | mobileOG_000002425 | transfer |
| bin479 | bin479_32 | mobileOG_000005363 | transfer |
| bin479 | bin479_32 | mobileOG_000015198 | transfer |
| bin479 | bin479_32 | mobileOG_000004680 | replication/recombination/repair |

Table S5. Catalog of mobile genetic elements (MGEs) encoded by *Enterococcus* spp. Metagenome-Assembled Genomes (MAGs).

| **Major mobileOG Category** | **bin161** | **bin381** | **bin436** |
| --- | --- | --- | --- |
| integration/excision | 46 | 12 | 11 |
| phage | 29 | 37 | 26 |
| replication/recombination/repair | 70 | 62 | 54 |
| stability/transfer/defense | 19 | 11 | 7 |
| transfer | 27 | 24 | 17 |
| total | 191 | 146 | 115 |

Table S6. ARGs encoded by *Enterococcus* spp. Metagenome-Assembled Genomes (MAGs).

| **Bin ID** | **Drug Class** | **Resistance Mechanism** | **AMR Gene Family** | **Gene** |
| --- | --- | --- | --- | --- |
| **Bin 161** | glycopeptide antibiotic | antibiotic target alteration | glycopeptide resistance gene cluster; vanT | vanT gene in vanG cluster |
|  | tetracycline antibiotic | antibiotic target protection | tetracycline-resistant ribosomal protection protein | tet(S) |
|  | glycopeptide antibiotic | antibiotic target alteration | vanY; glycopeptide resistance gene cluster | vanY gene in vanB cluster |
|  | lincosamide antibiotic | antibiotic inactivation | lincosamide nucleotidyltransferase (LNU) | lnuB |
|  | phenicol antibiotic | antibiotic efflux | major facilitator superfamily (MFS) antibiotic efflux pump | fexA |
| **Bin 381** | aminoglycoside antibiotic | antibiotic inactivation | AAC(6') | AAC(6')-Ii |
|  | macrolide antibiotic; fluoroquinolone antibiotic; rifamycin antibiotic | antibiotic efflux | ATP-binding cassette (ABC) antibiotic efflux pump | efrA |
|  | tetracycline antibiotic | antibiotic target protection | tetracycline-resistant ribosomal protection protein | tet(M) |
|  | glycopeptide antibiotic | antibiotic target alteration | vanY; glycopeptide resistance gene cluster | vanY gene in vanB cluster |
| **Bin 436** | glycopeptide antibiotic | antibiotic target alteration | glycopeptide resistance gene cluster; vanR | vanR gene in vanC cluster |
|  | glycopeptide antibiotic | antibiotic target alteration | glycopeptide resistance gene cluster; vanT | vanT gene in vanC cluster |
|  | glycopeptide antibiotic | antibiotic target alteration | glycopeptide resistance gene cluster; vanXY | vanXY gene in vanC cluster |
|  | glycopeptide antibiotic | antibiotic target alteration | glycopeptide resistance gene cluster; Van ligase | vanC |
|  | glycopeptide antibiotic | antibiotic target alteration | vanY; glycopeptide resistance gene cluster | vanY gene in vanB cluster |
|  | phosphonic acid antibiotic | antibiotic inactivation | fosfomycin thiol transferase | FosI |
|  | macrolide antibiotic; fluoroquinolone antibiotic; rifamycin antibiotic | antibiotic efflux | ATP-binding cassette (ABC) antibiotic efflux pump | efrA |

Table S7 Catalog of virulence factors encoded by the Metagenome-Assembled Genome (MAG) of *Enterococcus avium* (bin 161)

| **VFclass** | **Virulence factors** | **Related genes** | **bin161(Prediction)** |
| --- | --- | --- | --- |
|  |  |  | **draft (draft)** |
| Adherence | AS | -- | - |
|  |  | -- | - |
|  |  | asa1 | - |
|  |  | prgB/asc10 | - |
|  | Ace | ace | - |
|  | Acm | acm | - |
|  | Ebp pili | ebpA | - |
|  |  | ebpB | - |
|  |  | ebpC | - |
|  |  | srtC | - |
|  | EcbA | ecbA | orf00367; orf01453 |
|  | EfaA | efaA | orf02796 |
|  | Esp | esp | - |
|  | Scm | scm | - |
|  | SgrA | sgrA | - |
|  | D-alanine-polyphosphoribitol ligase (Listeria) | dltA | orf02657 |
|  | Streptococcal lipoprotein rotamase A (Streptococcus) | slrA | orf03439 |
| Antiphagocytosis | Capsule | cpsA/uppS | orf03861 |
|  |  | cpsB/cdsA | orf03862 |
|  |  | cpsC | - |
|  |  | cpsD | - |
|  |  | cpsE | - |
|  |  | cpsF | - |
|  |  | cpsG | - |
|  |  | cpsH | - |
|  |  | cpsI | - |
|  |  | cpsJ | - |
|  |  | cpsK | - |
|  | Capsule (Klebsiella) | -- | orf01279 |
|  |  | uge | orf01271 |
| Biofilm formation | BopD | bopD | orf02764 |
|  | Fsr locus | fsrA | - |
|  |  | fsrB | - |
|  |  | fsrC | - |
| Enzyme | Gelatinase | gelE | - |
|  | Hyaluronidase | -- | - |
|  |  | -- | - |
|  | SprE | sprE | - |
| Toxin | Cytolysin | cylA | - |
|  |  | cylB | - |
|  |  | cylI | - |
|  |  | cylL-l | - |
|  |  | cylL-s | - |
|  |  | cylM | - |
|  |  | cylR1 | - |
|  |  | cylR2 | orf00749; orf01898 |
| Cell surface components | Trehalose-recycling ABC transporter (Mycobacterium) | sugC | orf00265 |
| Immune evasion | Capsule (Staphylococcus) | -- | orf00601 |
|  | Capsule (Streptococcus) | -- | orf00536 |
|  |  | cps4I | orf00859 |
|  |  | rgpG | orf02027 |
|  | LOS (Campylobacter) | -- | orf02360 |
|  | Polysaccharide capsule (Bacillus) | epsE | orf00853; orf01285 |
| Phagosome arresting | Nucleoside diphosphate kinase (Mycobacterium) | ndk | orf00328 |
| Protease | Serine protease (Streptococcus) | htrA/degP | orf00548 |
| Regulation | LisR/LisK (Listeria) | lisR | orf00656 |
| Surface protein anchoring | Lipoprotein diacylglyceryl transferase (Listeria) | lgt | orf02230 |

Table S8 Catalog of virulence factors encoded by the Metagenome-Assembled Genome (MAG) of *Enterococcus thailandicus* (bin 381)

| **VFclass** | **Virulence factors** | **Related genes** | **bin381(Prediction)** |
| --- | --- | --- | --- |
|  |  |  | **draft (draft)** |
| Adherence | AS | -- | - |
|  |  | -- | - |
|  |  | asa1 | - |
|  |  | prgB/asc10 | - |
|  | Ace | ace | - |
|  | Acm | acm | - |
|  | Ebp pili | ebpA | orf02320 |
|  |  | ebpB | orf02321 |
|  |  | ebpC | orf02322 |
|  |  | srtC | orf02323; orf02350 |
|  | EcbA | ecbA | - |
|  | EfaA | efaA | orf00063 |
|  | Esp | esp | - |
|  | Scm | scm | - |
|  | SgrA | sgrA | - |
|  | Fibronectin-binding proteins (Streptococcus) | pavA | orf02523 |
| Antiphagocytosis | Capsule | cpsA/uppS | orf01840 |
|  |  | cpsB/cdsA | orf01841 |
|  |  | cpsC | orf00291 |
|  |  | cpsD | - |
|  |  | cpsE | - |
|  |  | cpsF | - |
|  |  | cpsG | - |
|  |  | cpsH | - |
|  |  | cpsI | - |
|  |  | cpsJ | - |
|  |  | cpsK | - |
| Biofilm formation | BopD | bopD | orf00935 |
|  | Fsr locus | fsrA | - |
|  |  | fsrB | - |
|  |  | fsrC | - |
| Enzyme | Gelatinase | gelE | - |
|  | Hyaluronidase | -- | orf00554 |
|  |  | -- | - |
|  | SprE | sprE | - |
|  | Serine-threonine phosphatase (Listeria) | stp | orf01431 |
|  | Streptococcal enolase (Streptococcus) | eno | orf02254 |
| Toxin | Cytolysin | cylA | - |
|  |  | cylB | - |
|  |  | cylI | - |
|  |  | cylL-l | - |
|  |  | cylL-s | - |
|  |  | cylM | - |
|  |  | cylR1 | - |
|  |  | cylR2 | orf01377 |
| Immune evasion | Capsule (Staphylococcus) | capP | orf01524 |
|  | Capsule (Streptococcus) | -- | orf00302 |
| Iron uptake | Periplasmic binding protein-dependent ABC transport systems (Vibrio) | vctC | orf00708 |
| Magnesium uptake | Mg2+ transport (Salmonella) | mgtB | orf00113 |
| Surface protein anchoring | Lipoprotein diacylglyceryl transferase (Listeria) | lgt | orf01157 |

Table S9 Catalog of virulence factors encoded by the Metagenome-Assembled Genome (MAG) of *Enterococcus casseliflavus* (bin 436)

| **VFclass** | **Virulence factors** | **Related genes** | **bin436(Prediction)** |
| --- | --- | --- | --- |
|  |  |  | **draft (draft)** |
| Adherence | AS | -- | - |
|  |  | -- | - |
|  |  | asa1 | - |
|  |  | prgB/asc10 | - |
|  | Ace | ace | - |
|  | Acm | acm | - |
|  | Ebp pili | ebpA | orf01810 |
|  |  | ebpB | - |
|  |  | ebpC | orf01809 |
|  |  | srtC | orf01808 |
|  | EcbA | ecbA | - |
|  | EfaA | efaA | orf02448 |
|  | Esp | esp | - |
|  | Scm | scm | - |
|  | SgrA | sgrA | - |
|  | Fibronectin-binding proteins (Streptococcus) | pavA | orf03073 |
|  | Streptococcal lipoprotein rotamase A (Streptococcus) | slrA | orf02103 |
| Antiphagocytosis | Capsule | cpsA/uppS | orf01890 |
|  |  | cpsB/cdsA | orf01889 |
|  |  | cpsC | - |
|  |  | cpsD | - |
|  |  | cpsE | - |
|  |  | cpsF | - |
|  |  | cpsG | - |
|  |  | cpsH | - |
|  |  | cpsI | - |
|  |  | cpsJ | orf01831 |
|  |  | cpsK | - |
| Biofilm formation | BopD | bopD | orf01315 |
|  | Fsr locus | fsrA | - |
|  |  | fsrB | - |
|  |  | fsrC | - |
| Enzyme | Gelatinase | gelE | - |
|  | Hyaluronidase | -- | - |
|  |  | -- | - |
|  | SprE | sprE | - |
| Toxin | Cytolysin | cylA | - |
|  |  | cylB | - |
|  |  | cylI | - |
|  |  | cylL-l | - |
|  |  | cylL-s | - |
|  |  | cylM | - |
|  |  | cylR1 | - |
|  |  | cylR2 | - |
| Copper uptake | Copper exporter (Mycobacterium) | ctpV | orf00711 |
| Immune evasion | Capsule (Staphylococcus) | capL | orf02554 |
|  | Capsule (Streptococcus) | -- | orf00446 |
|  |  | cps4I | orf01599 |
|  |  | cpsY | orf00986 |
|  | Polysaccharide capsule (Bacillus) | -- | orf02543 |
|  |  | epsE | orf02545 |
| Invasion | Flagella (Burkholderia) | fliP | orf00677 |
| Nutritional factor | Allantoin utilization (Klebsiella) | -- | orf03262 |
|  |  | -- | orf03260 |
| Protease | Serine protease (Streptococcus) | htrA/degP | orf03189 |
| Regulation | CheA/CheY (Listeria) | cheY | orf00693 |
| Secretion system | T2SS (Type II secretion system) (Shigella) | gspE | orf02603 |
|  | Type III secretion system (Bordetella) | bscN | orf00668 |
| Surface protein anchoring | Lipoprotein diacylglyceryl transferase (Listeria) | lgt | orf02748 |

Table S10 Comparison of persistent organic pollutants (POPs) biodegradation extents in soil/sediment matrices from representative studies.

| Pollutant | Matrix | Degradation Strategy | Degradation Extent | Time | Reference |
| --- | --- | --- | --- | --- | --- |
| **γ-HCH** | **Soil** | **Bioaugmentation with constructed consortium** | **37.0% – 43.9%** | **30 d** | **This study** |
| **γ-HCH** | **Liquid culture** | **Constructed consortium** | **67.3% – 99.3%** | **10 d** | **This study** |
| γ-HCH | Soil | Bioaugmentation with degrading consortium | 15.8% | 28 d | (Cheng et al., 2024) |
| γ-HCH | Soil | Bioaugmentation + exogenous addition of conductive material | 34.5% – 67.2% | 28 d | (Cheng et al., 2024) |
| PCB101 | Soil | Ferric oxide nanomaterials and plant-rhizobacteria symbionts cogenerate iron plaque | 23.2% | 120 d | (Zheng et al., 2024) |
| Total PCBs | Soil | Bioaugmentation with resuscitated strain | 56.7% | 28 d | (Zhou et al., 2023) |
| PCBs | Soil | Bioaugmentation with *Pleurotus ostreatus* | 18.5% – 50.5% | 12 weeks | (Stella et al., 2017) |
| Biocide | Wood-soil mixture | Bioaugmentation with defined soil bacterial community | 20% | 105 d | (Jakobs-Schönwandt et al., 2010) |

Table S11. Comparison of γ‑HCH degradation rates in liquid culture and sediment microcosms.

| **Consortium** | Average liquid rate (% d⁻¹) | Average sediment rate (% d⁻¹) | Rate retention (%) |
| --- | --- | --- | --- |
| **M** | 6.73 | 1.23 | 18.3 |
| **Y13** | 9.93 | 1.46 | 14.7 |

Table S12. Summary of multi-level evidence supporting the potential involvement of pceA-like reductive dehalogenases in anaerobic γ-HCH degradation.

| **Line of evidence** | **Key finding** | **Reference** |
| --- | --- | --- |
| **Environmental detection** | *pceA* abundance correlates with lindane degradation in flooded paddy soils. | (Chuang et al., 2020) |
| **Microbial functional validation (1)** | *Dehalobacter* achieves complete lindane biodegradation in anaerobic consortia. | (Puentes Jácome et al., 2021) |
| **Microbial functional validation (2)** | *Dehalobacter* sp. reductively dechlorinates β-HCH in coculture. | *(van Doesburg et al., 2005).* |
| **Microbial functional validation (3)** | *Dehalobacter*-containing culture dechlorinates all chlorobenzene congeners. | (Cao et al., 2025) |
| **Molecular mechanism (structural)** | PceA crystal structure reveals B12-dependent scaffold with broad substrate-binding region. | (Bommer et al., 2014) |
| **Molecular mechanism (catalytic)** | B12-dependent reductive dehalogenases catalyze dehalorespiration across diverse aromatic halides; theoretical models predict substrate reactivity. | (Zhang et al., 2023) |

Table S13. Summary of reported effects of DMSO on microbial metabolism.

| **Experimental system** | **DMSO concentration (v/v)** | **effect** | **refence** |
| --- | --- | --- | --- |
| Bioreactors | > 1% | Affect partial denitrification | Wang et al., 2024 |
| laboratory  scale sequencing batch reactors | > 2% | toxic to microorganisms | Wang et al., 2023 |
| Soil bacterial communities | > 5% | affect the degree of bacterial growth inhibition | Modrzyński et al., 2019 |
| *Saccharomyces cerevisiae* yeast | 2–20% | little effect on the metabolic activity and the stability of their cell membranes | Święciło et al., 2024 |
| Liquid system | 0.04% | - | This study |
| Sediment microcosms | 0.004% | - | This study |

Table S14. Summary of overall statistical analyses for figure panels with group comparisons.

| **Figure** | **Panel** | **Variable** | **Groups compared** | **Statistical test** | **Overall *p*‑value** |
| --- | --- | --- | --- | --- | --- |
| Fig. 1 | a | γ‑HCH concentration (C/C₀) | CK, S, M, Y13 | One‑way ANOVA | 0.01 |
| Fig. 1 | b | pH variation (ΔpH) | CK, S, M, Y13 | One‑way ANOVA | 6.11 × 10⁻⁵ |
| Fig. 1 | c | Shannon diversity index | M, S, Y13 | One‑way ANOVA | 0.02 |
| Fig. 1 | d | Average variation degree (AVD) | M, S, Y13 | One‑way ANOVA | 0.03 |
| Fig. 1 | e | Modified stochasticity ratio (MST) | M, S, Y13 | One‑way ANOVA | 0.03 |
| Fig. 5 | d | Total ARG abundance | M, S, Y13 | One‑way ANOVA | 0.002 |
| Fig. 5 | e | ARG count | M, S, Y13 | One‑way ANOVA | 0.008 |
| Fig. 6 | b | CO₂‑reductive methanogenesis pathway abundance | M, S, Y13 | One‑way ANOVA | 4.73 × 10⁻⁴ |
| Fig. 6 | c | CH₄/CO₂ ratio | CK, S, M, Y13 | One‑way ANOVA | 0.003 |

Table S15. Average nucleotide identity (ANI) matrix of *Enterococcus* genomes analyzed in this study.

|  | ***Enterococcus avium* ATCC 14025** | ***Enterococcus casseliflavus* ATCC 49996** | ***Enterococcus thailandicus* DSM 21767** | **PseS3** | **R1** | **R12** | **R3** | **bin161** | **bin381** | **bin436** |
| --- | --- | --- | --- | --- | --- | --- | --- | --- | --- | --- |
| ***Enterococcus avium* ATCC 14025** | ***100.0*** | 69.5 | 70.6 | ***98.0*** | 70.8 | 69.2 | 70.7 | ***98.2*** | 70.5 | 69.1 |
| ***Enterococcus casseliflavus* ATCC 49996** | 69.5 | ***100.0*** | 70.5 | 70.0 | 70.6 | ***98.2*** | 70.7 | 69.2 | 70.1 | ***98.2*** |
| ***Enterococcus thailandicus* DSM 21767** | 70.6 | 70.5 | ***100.0*** | 71.4 | ***99.3*** | 70.6 | ***99.3*** | 71.2 | ***99.3*** | 70.3 |
| **PseS3** | ***98.0*** | 70.0 | 71.4 | ***100.0*** | 71.5 | 69.7 | 73.7 | ***100.0*** | 70.4 | 69.2 |
| **R1** | 70.8 | 70.6 | ***99.3*** | 71.5 | ***100.0*** | 70.5 | ***100.0*** | 70.4 | ***100.0*** | 69.9 |
| **R12** | 69.2 | ***98.2*** | 70.6 | 69.7 | 70.5 | ***100.0*** | 70.5 | 69.1 | 69.9 | ***100.0*** |
| **R3** | 70.7 | 70.7 | ***99.3*** | 73.7 | ***100.0*** | 70.5 | ***100.0*** | 70.4 | ***100.0*** | 69.9 |
| **bin161** | ***98.2*** | 69.2 | 71.2 | ***100.0*** | 70.4 | 69.1 | 70.4 | ***100.0*** | 70.2 | 69.2 |
| **bin381** | 70.5 | 70.1 | ***99.3*** | 70.4 | ***100.0*** | 69.9 | ***100.0*** | 70.2 | ***100.0*** | 69.9 |
| **bin436** | 69.1 | ***98.2*** | 70.3 | 69.2 | 69.9 | ***100.0*** | 69.9 | 69.2 | 69.9 | ***100.0*** |

**References**

Bommer M, Kunze C, Fesseler J, et al. 2014. Structural basis for organohalide respiration. Science [J], 346: 455-458.

Bowers R M, Kyrpides N C, Stepanauskas R, et al. 2017. Minimum information about a single amplified genome (MISAG) and a metagenome-assembled genome (MIMAG) of bacteria and archaea. Nature Biotechnology [J], 35: 725-731.

Cao S, Tang D, Tian Y, et al. 2025. Anaerobic reductive dechlorination of chlorobenzenes by an enrichment culture containing Dehalobacter species. International Biodeterioration & Biodegradation [J], 201.

Chen S, Zhou Y, Chen Y, et al. 2018. fastp: an ultra-fast all-in-one FASTQ preprocessor. Bioinformatics [J], 34: i884-i890.

Cheng J, Wu J, Su X, et al. 2024. Conductive material and AHLs addition altered soil microbiome and facilitated γ-HCH dechlorination but inhibited CH4 cumulation. Soil Biology and Biochemistry [J], 191.

Chklovski A, Parks D H, Woodcroft B J, et al. 2023. CheckM2: a rapid, scalable and accurate tool for assessing microbial genome quality using machine learning. Nature Methods [J], 20: 1203-1212.

Chuang S, Wang B, Chen K, et al. 2020. Microbial catabolism of lindane in distinct layers of acidic paddy soils combinedly affected by different water managements and bioremediation strategies. Science of the Total Environment [J], 746.

Jakobs-Schönwandt D s e, Mathies H, Abraham W-R, et al. 2010. Biodegradation of a Biocide (Cu-N-Cyclohexyldiazenium Dioxide) Component of a Wood Preservative by a Defined Soil Bacterial Community. Applied and Environmental Microbiology [J], 76: 8076-8083.

Kang D D, Li F, Kirton E, et al. 2019. MetaBAT 2: an adaptive binning algorithm for robust and efficient genome reconstruction from metagenome assemblies. PeerJ [J], 7: e7359.

Li D, Liu C M, Luo R, et al. 2015. MEGAHIT: an ultra-fast single-node solution for large and complex metagenomics assembly via succinct de Bruijn graph. Bioinformatics [J], 31: 1674-1676.

Liu M, Su X, Yuan J, et al. 2025. Unravelling the processes involved in biodegradation of chlorinated organic pollutant: From microbial community to isolated organohalide degraders. Water Research [J]: 122730.

Martin M 2011. Cutadapt removes adapter sequences from high-throughput sequencing reads. EMBnet.journal [J], 17: 10-12.

Nissen J N, Johansen J, Allesøe R L, et al. 2021. Improved metagenome binning and assembly using deep variational autoencoders. Nature Biotechnology [J], 39: 555-560.

Olm M R, Brown C T, Brooks B, et al. 2017. dRep: a tool for fast and accurate genomic comparisons that enables improved genome recovery from metagenomes through de-replication. The ISME Journal [J], 11: 2864-2868.

Pan S, Zhu C, Zhao X-M, et al. 2022. A deep siamese neural network improves metagenome-assembled genomes in microbiome datasets across different environments. Nature Communications [J], 13.

Puentes Jácome L A, Lomheim L, Gaspard S, et al. 2021. Biodegradation of Lindane (γ-Hexachlorocyclohexane) To Nontoxic End Products by Sequential Treatment with Three Mixed Anaerobic Microbial Cultures. Environmental Science & Technology [J], 55: 2968-2979.

Stella T, Covino S, Čvančarová M, et al. 2017. Bioremediation of long-term PCB-contaminated soil by white-rot fungi. Journal of Hazardous Materials [J], 324: 701-710.

Uritskiy G V, DiRuggiero J, Taylor J 2018. MetaWRAP—a flexible pipeline for genome-resolved metagenomic data analysis. Microbiome [J], 6.

van Doesburg W, van Eekert M H A, Middeldorp P J M, et al. 2005. Reductive dechlorination of β-hexachlorocyclohexane (β-HCH) by a Dehalobacter species in coculture with a Sedimentibacter sp. FEMS Microbiology Ecology [J], 54: 87-95.

Zhang S, Wen W, Xia X, et al. 2023. Insight into the Mechanism Underlying Dehalococcoides mccartyi Strain CBDB1-Mediated B12-Dependent Aromatic Reductive Dehalogenation. Environmental Science & Technology [J], 57: 10773-10781.

Zheng T, Hou J, Wu T, et al. 2024. Ferric Oxide Nanomaterials and Plant-Rhizobacteria Symbionts Cogenerate Iron Plaque for Removing Highly Chlorinated Contaminants in Dryland Soils. Environmental Science & Technology [J], 58: 11063-11073.

Zhou X, Zhang S, Wang R, et al. 2023. A novel strategy for enhancing bioremediation of polychlorinated biphenyl-contaminated soil with resuscitation promoting factor and resuscitated strain. Journal of Hazardous Materials [J], 447.

Modrzyński J J, Christensen J H, Brandt K K 2019. Evaluation of dimethyl sulfoxide (DMSO) as a co-solvent for toxicity testing of hydrophobic organic compounds. Ecotoxicology [J], 28: 1136-1141.

Święciło A, Januś E, Krzepiłko A, et al. 2024. The effect of DMSO on Saccharomyces cerevisiae yeast with different energy metabolism and antioxidant status. Scientific Reports [J], 14.

Wang Z, Zhang J, Zhou J, et al. 2023. Dosage-Dependent Toxicity of Universal Solvent Dimethyl Sulfoxide to the Partial Nitrification Wastewater Treatment Process. ACS ES&T Water [J], 3: 848-856.

Wang Z-B, Zhang J, Miao Q, et al. 2024. Achieving Stable Partial Denitrification by Selective Inhibition of Nitrite Reductase with the Biosafe Aprotic Solvent DMSO. Environmental Science & Technology [J], 58: 21242-21250.
